# Differential effects of Δ9-tetrahydrocannabinol dosing on correlates of schizophrenia in the sub-chronic PCP rat model

**DOI:** 10.1371/journal.pone.0230238

**Published:** 2020-03-12

**Authors:** Alexandre Seillier, Alex A. Martinez, Andrea Giuffrida

**Affiliations:** Department of Pharmacology, University of Texas Health Science Center at San Antonio, San Antonio, Texas, United States of America; Hudson Institute, AUSTRALIA

## Abstract

Social withdrawal in the sub-chronic phencyclidine (PCP) rat model, a behavioral correlate of the negative symptoms of schizophrenia, results from deficits in brain endocannabinoid transmission. As cannabis intake has been shown to affect negatively the course and expression of psychosis, we tested whether the beneficial effects of endocannabinoid-mediated CB_1_ activation on social withdrawal in PCP-treated rats (5 mg/kg, twice daily for 7 days)also occurred after administration of Δ9-tetrahydrocannabinol (THC; 0.1, 0.3, 1.0 mg/kg, i.p.). In addition, we assessed whether THC affected two correlates of positive symptoms: 1) motor activity induced by *d*-amphetamine (0.5 mg/kg, i.p.), and 2) dopamine neuron population activity in the ventral tegmental area (VTA). After the motor activity test, the brains from *d*-amphetamine-treated animals were collected and processed for measurements of endocannabinoids and activation of Akt/GSK3β, two molecular markers involved in the pathophysiology of schizophrenia. In control rats, THC dose-dependently produced social interaction deficits and aberrant VTA dopamine neuron population activity similar to those observed in PCP-treated animals. In PCP-treated rats, only the lowest dose of THC reversed PCP-induced deficits, as well as PCP-induced elevation of the endocannabinoid anandamide (AEA) in the nucleus accumbens. Last, THC activated the Akt/GSK3β pathway dose-dependently in both control and PCP-treated animals. Taken together, these data suggest that only low doses of THC have beneficial effects on behavioral, neurochemical and electrophysiological correlates of schizophrenia symptoms. This observation may shed some light on the controversial hypothesis of marijuana use as self-medication in schizophrenic patients.

## Introduction

*Cannabis sativa* is the most common illicit drug used by schizophrenic patients [1,2], who consume it at higher rates than the general population [3]. Although the evidence for an association between cannabis use and schizophrenia is compelling [4,5], the precise nature of this relationship remains a matter of debate [5–7]. Several hypotheses have been formulated in this regard, specifically: 1) the ‘diathesis-stress model’, which considers cannabis as a contributing cause to schizophrenia in vulnerable individuals; 2) the ‘shared vulnerability’ hypothesis, which implies the existence of a third causal factor (e.g. genetic susceptibility for both schizophrenia and cannabis use disorder); and 3) the ‘self-medication’ hypothesis, which points to cannabis intake as a way to cope with prodromal symptoms and/or side effects associated with antipsychotic treatments (reversed causality). Nevertheless, no hypothesis alone seems to adequately capture the complexity of the link between cannabis and schizophrenia. The ability of cannabis consumption to induce psychotomimetic symptoms has long been recognized [5] and attributed to its psychoactive ingredient, Δ9-tetrahydrocannabinol (THC). The commonly accepted view is that cannabis exposure, as in the case of other drugs of abuse [8], has a negative impact on the disease outcome [5] leading to: worsening of psychotic symptoms [9,10], relapse [11,12], and decreased global functioning over time ([10], but see [13]). However, cannabis may differently affect the clinical features of schizophrenia depending on the type of symptoms. Indeed, a large body of literature has consistently linked cannabis consumption to the exacerbation of positive symptoms [14] despite some conflicting findings [1]. On the other hand, cannabis use has been shown to ameliorate negative symptoms [1,15], although not consistently [12]. Concerning the effects of THC on cognitive deficits, different research groups found them to be worsened [16], unaffected [17], or even improved [13,18–20].

The ‘cannabinoid hypothesis’ of schizophrenia postulates that overactivity of the endocannabinoid system contributes to the pathogenesis of the disease [21]. However, new experimental evidence has challenged this view [22–26] and suggested that CB_1_ abnormalities may vary between specific disease subtypes [27–30]. For instance, Dalton et al. [28] showed that only paranoid schizophrenics had higher CB_1_ receptor levels in the dorsolateral prefrontal cortex, whereas other subgroups had lower CB_1_ densities, as previously reported by Eggan et al. [22]. On the same line, Wong et al. [30] reported that CB_1_ receptor binding is positively correlated with the severity of positive symptoms, whereas patients with reduced CB_1_ binding had more pronounced negative symptomatology. Thus, the role played by the endocannabinoid system in schizophrenia may vary greatly depending on the specific diagnosis and/or type of schizophrenic symptoms [31]. To add a further level of complexity, cannabis exposure also produces different effects in healthy versus schizophrenic subjects [18,19]. Similar observations have been reported in preclinical settings as well [32–34]. The diverging effects of cannabinoids may be attributed to pre-existing dysfunctions of the endocannabinoid system in the animal models considered. For instance, in the sub-chronic phencyclidine (PCP) rat model of schizophrenia, we previously demonstrated that social withdrawal–a core negative symptoms of schizophrenia–was associated with deficient endocannabinoid-mediated CB_1_ activation [33,35]. On the other hand, the working memory deficit in the same model was linked to increased activity at CB_1_ receptors [33]. In agreement with these observations, systemic administration of the endocannabinoid-enhancing drug URB597 reversed PCP-induced social withdrawal, but had no effect on PCP-induced deficit in working memory [33], which was reversed instead by CB_1_ receptor antagonism [33]. Interestingly, the same pharmacological treatments in control animals produced behavioral deficits similar to those induced by PCP [33,35]. In line with our findings, Spano et al. [34] showed that chronic administration of the cannabinoid agonist WIN55,212–2 attenuated PCP-induced deficits in sociability, but caused social withdrawal in saline-treated controls. Despite these observations, it is still unknown whether THC would alleviate or exacerbate schizophrenia-like symptoms in adult animal models (with pre-existing deficits), especially in view of the fact that THC might disrupt, rather than enhance, endocannabinoid transmission [36].

In this study, we tested whether THC had beneficial effects on social withdrawal in PCP-treated rats as those reported with endocannabinoid-enhancing drugs [35,37]. We also assessed the effects of THC on: 1) motor hyperactivity induced by *d*-amphetamine [38], and 2) dopamine neuron population overactivity in the ventral tegmental area (VTA; [39]), two correlates of positive-like symptoms, as well as 3) endocannabinoid levels in the nucleus accumbens [33,35] and 4) Akt/GSK3β signaling (as this pathway has been involved in the pathogenesis of schizophrenia [40]). Our data suggest that low, but not high, doses of THC have beneficial effects on several correlates of schizophrenia symptoms.

## Results

### Dose-dependent effects of THC in saline and PCP-treated animals

**Fig 1:**
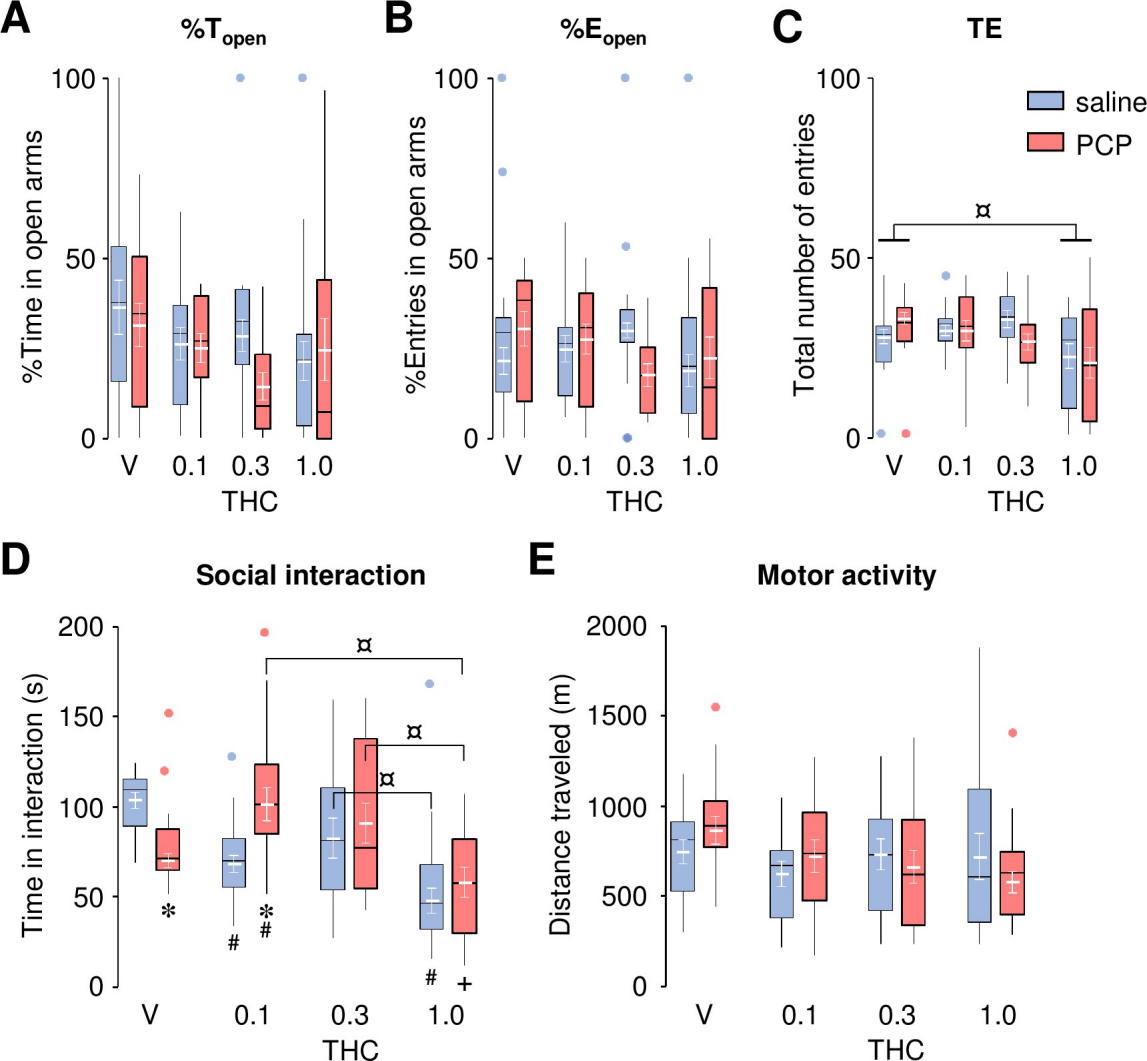
Effects of THC (0.1–1.0 mg/kg) on anxiety-like behavior, social interaction and motor activity in saline- and PCP- treated rats. Percent time spent in the open arms (%T_open_; **A**), percent of open arms entries (%E_open_; **B**) and total number of entries (TE; **C**) in the EPM task. Time spent in social interaction (**D**). Distance traveled in the Actimot activity box following *d*-amphetamine (0.5 mg/kg, i.p.) administration (**E**). The raw data for this figure are reported in the supplemental file (S1 Raw data) and are summarized here as boxplots computed using Carling’s modification [42]; outliers are depicted as blue (saline) or red (PCP) circles. Values (in white) are expressed as mean ± S.E.M. (n = 10–14 per group). * *P* < 0.05 compared to saline-treated controls, # *P* < 0.05 compared to vehicle (V) controls, + *P* < 0.05 compared to saline-treated vehicle (V) controls, ¤ *P* < 0.05 (Newman-Keuls *post-hoc* test).

PCP-treated rats did not differ from saline controls in any of the Elevated Plus Maze (EPM) variables (%T_open_, %E_open_ and TE; Fig 1A, 1B and 1C, respectively). Systemic administration of THC (0.1–1.0 mg/kg) did not significantly affect this profile, except for a slight decrease in the total number of entries (TE) after the highest dose of THC (1 mg/kg; *P* = 0.008 compared to vehicle control). ANOVA revealed no main effect of Group (*F*_1,102_ = 1.06, *P* = 0.30, *F*_1,97_ = 0.05, *P* = 0.82 and *F*_1,101_ = 0.16, *P* = 0.69; for %T_open_, %E_open_ and TE, respectively), Drug (*F*_3,102_ = 1.81, *P* = 0.15 and *F*_1,97_ = 0.77, *P* = 0.51; for %T_open_ and %E_open_, respectively), nor interaction between these two factors (*F*_3,102_ = 0.78, *P* = 0.51, *F*_1,97_ = 2.12, *P* = 0.10 and *F*_1,101_ = 1.44, *P* = 0.24; for %T_open_, %E_open_ and TE, respectively), with the exception of a main effect of Drug for the total number of entries (TE; *F*_1,101_ = 4.73, *P* = 0.004).

As previously reported [41], PCP-treated rats spent significantly less time interacting with their conspecific (Fig 1D), a behavioral phenotype reminiscent of negative symptoms. THC reversed PCP-induced social withdrawal at the lowest (0.1 mg/kg), but not at the highest (1.0 mg/kg) dose, whereas it significantly reduced social interaction in saline-treated rats at both doses. ANOVA revealed no main effect of Group (*F*_1,99_ = 0.63, *P* = 0.43), but a significant effect of Drug (*F*_3,99_ = 8.37, *P* < 0.001), and a significant interaction between these two factors (*F*_3,99_ = 5.64, *P* = 0.001).

In the motor activity test, systemic administration of THC (0.1–1.0 mg/kg) did not affect the distance traveled in a novel environment (i.e. during the 30-min habituation period) in either saline- or PCP-treated animals (S1 Fig). *D*-amphetamine (0.5 mg/kg, i.p.) significantly increased the distance traveled by the animals (i.e. compared to the habituation period), but PCP-treated rats failed to show enhanced *d*-amphetamine-induced hyperactivity (i.e. a behavioral phenotype reminiscent of positive symptoms) compared to saline-treated controls. Furthermore, THC did not affect the distance traveled in response to *d*-amphetamine in either saline- or PCP-treated animals (Fig 1E). ANOVA did not reveal any main effect of Group (*F*_1,102_ = 0.00, *P* = 0.99), Drug (*F*_3,102_ = 1.27, *P* = 0.29), or interaction between these two factors (*F*_3,102_ = 1.07, *P* = 0.37).

**Fig 2:**
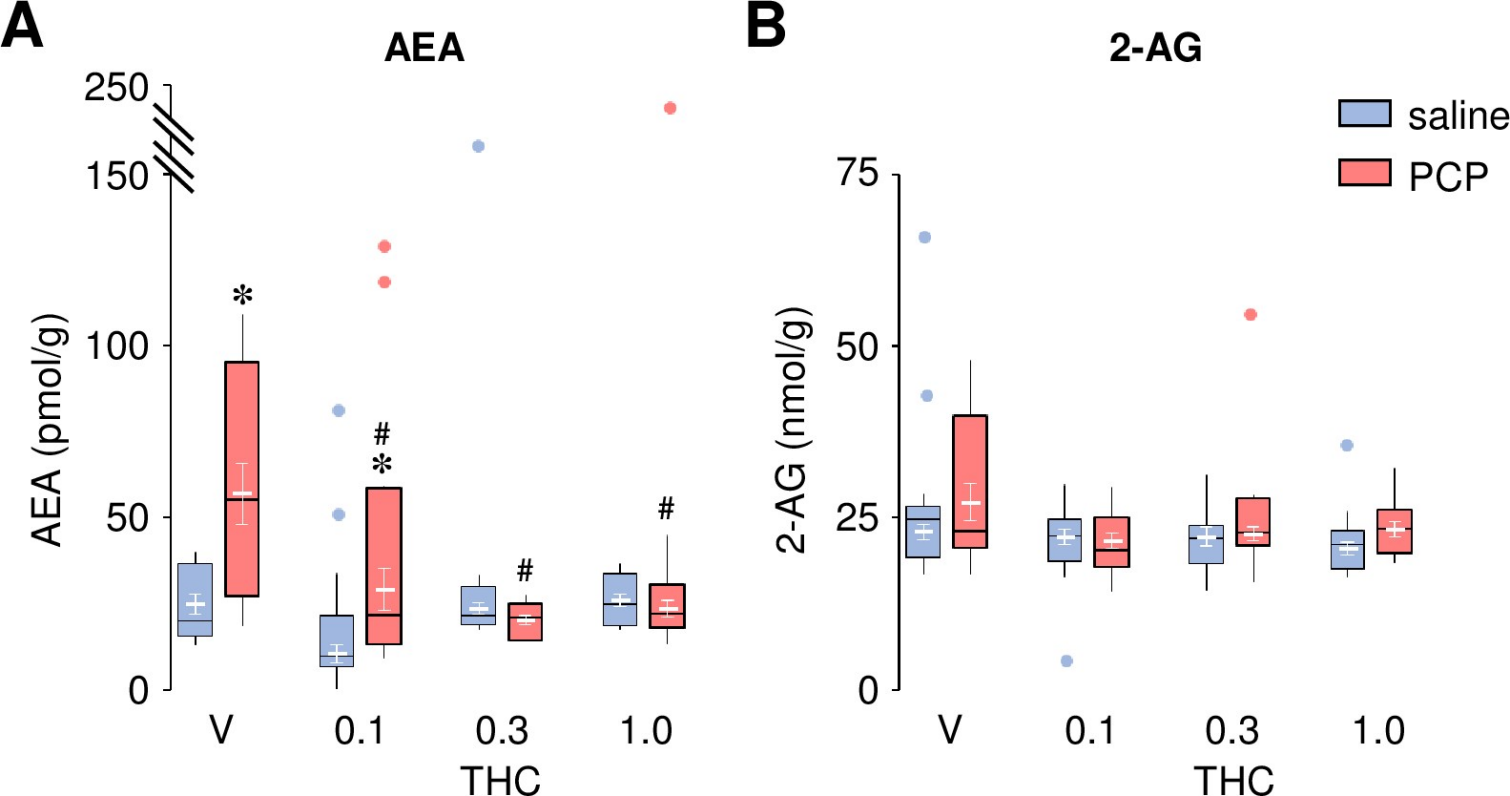
THC reversed PCP-induced increase in AEA levels in the nucleus accumbens. Effects of THC (0.1–1.0 mg/kg) on AEA (**A**) and 2-AG (**B**) levels in the nucleus accumbens of saline- and PCP- treated rats immediately after the motor activity task (*d*-amphetamine-induced hyperactivity; see Fig 1E). The raw data for this figure are reported in the supplemental file (S1 Raw data) and are summarized here as boxplots computed using Carling’s modification [42]; outliers are depicted as blue (saline) or red (PCP) circles. Values (in white) are expressed as mean ± S.E.M. (n = 11–14 per group). * *P* < 0.05 compared to saline-treated controls, # *P* < 0.05 compared to vehicle (V) controls (Newman-Keuls *post-hoc* test).

As elevated anandamide (AEA) has been observed in the cerebrospinal fluid of acute paranoid schizophrenics [24,26,43]–a condition accompanied by striatal hyperdopaminergia [44]–we assessed the effects of THC on endocannabinoid levels immediately after the motor activity test (i.e. in the presence of *d*-amphetamine). In PCP-treated animals, we observed increased AEA levels in the nucleus accumbens; this increase was fully reversed by THC (Fig 2A). ANOVA revealed a main effect of Group (*F*_1,96_ = 14.01, *P* = 0.0003), Drug (*F*_3,96_ = 10.56, *P* < 0.001), and interaction between these two factors (*F*_3,96_ = 8.50, *P* < 0.001). In contrast, 2-arachidonyl glycerol (2-AG) levels were not significantly altered in any experimental group irrespective of the presence of THC (Fig 2B; ANOVA, Group: *F*_1,97_ = 2.83, *P* = 0.096; Drug: *F*_3,97_ = 2.16, *P* = 0.098; Drug X Group: *F*_3,97_ = 1.16, *P* = 0.33).

**Fig 3:**
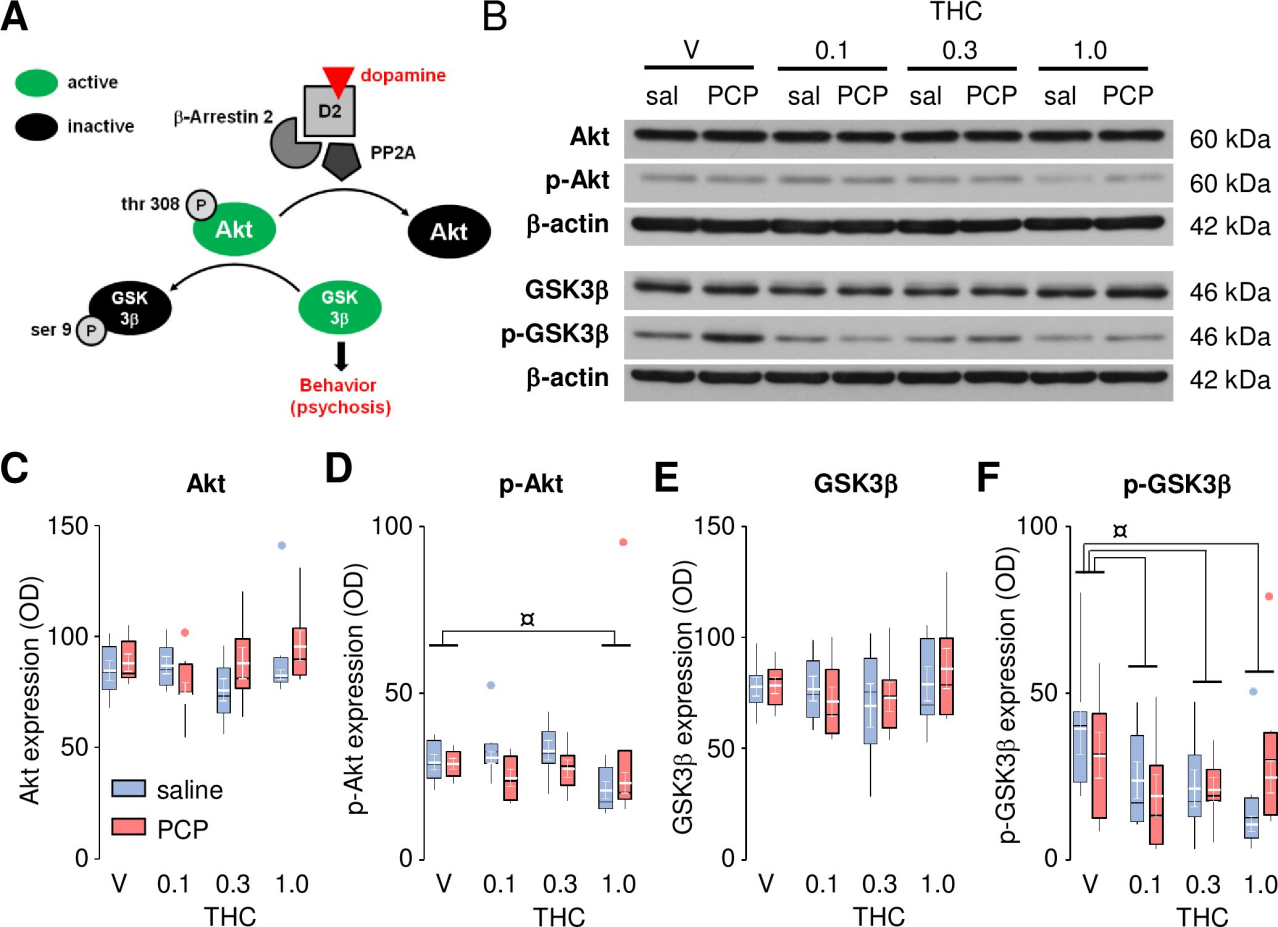
Effects of THC (0.1–1.0 mg/kg) in saline- and PCP- treated rats on the Akt-GSK3β pathway. Scheme of the Akt/GSK-3β signaling pathway and the sequence of events leading to the expression of dopamine-associated behaviors (based on [45]; **A**). Representative western blot data (**B**) showing Akt expression (normalized to β-actin; **C**) and phosphorylation (p-Akt at thr 308; normalized to Akt; **D**), as well as GSK3β expression (normalized to β-actin; **E**) and phosphorylation (p-GSK3β at ser 9; normalized to GSK3β; **F**) in saline- (sal) and PCP-treated rats. The raw data for this figure are reported in the supplemental file (S1 Raw data) and are summarized here as boxplots computed using Carling’s modification [42]; outliers are depicted as blue (saline) or red (PCP) circles. Values (in white) are expressed as mean ± S.E.M. (n = 6–7 per group). ¤ *P* < 0.05 (Newman-Keuls *post-hoc* test).

It has been proposed that the Akt/GSK-3β signaling pathway might be involved in the pathogenesis of schizophrenia [40] and in the expression of dopamine-associated behaviors [45], and that antipsychotic medication may may exert their beneficial effects, at least in part, by modulating this pathway [40]. Specifically, Akt1 levels and Akt-dependent phosphorylation of GSK3β (at Ser9) were reduced in the frontal cortex of schizophrenic patients. Given that activation of dopamine D_2_ receptors led to the dephosphorylation of Akt (at Thr 308), resulting in its inactivation and the consequent suppression of its inhibitory activity on GSK3β (see Fig 3A), we investigated the phosphorylation state of Akt1 (at Thr 308) and GSK3β (at Ser 9) to assess their possible activation immediately after the motor activity test. Western blot analysis (Fig 3B) showed that Akt1 and GSK3β protein levels were similar in saline- and PCP-treated animals and not affected by THC administration (Fig 3C and 3E). Phosphorylated Akt1 (p-Akt1) and GSK3β (p-GSK3β) did not differ between saline- and PCP-treated rats (Fig 3B, 3D and 3E). However, p-Akt1 was decreased by the highest dose of THC (1 mg/kg; Fig 3D), and p-GSK3β by all doses (Fig 3E). For Akt1 and GSK3β, ANOVA revealed no main effect of Group (*F*_1,46_ = 1.28, *P* = 0.26 and *F*_1,48_ = 0.07, *P* = 0.79, respectively), Drug (*F*_3,46_ = 1.15, *P* = 0.34 and *F*_3,48_ = 1.03, *P* = 0.39, respectively), nor interaction between these two factors (*F*_3,46_ = 2.61, *P* = 0.06 and *F*_3,48_ = 0.31, *P* = 0.82, respectively). For p-Akt1 and p-GSK3β, ANOVA revealed no main effect of Group (*F*_1,46_ = 1.96, *P* = 0.17 and *F*_1,46_ = 0.00, *P* = 0.95, respectively) nor interaction between the two factors (*F*_3,46_ = 1.23, *P* = 0.31 and *F*_3,46_ = 1.35, *P* = 0.27, respectively), but a significant Drug effect (*F*_3,46_ = 4.00, *P* = 0.01 and *F*_3,46_ = 3.67, *P* = 0.02, respectively).

**Fig 4:**
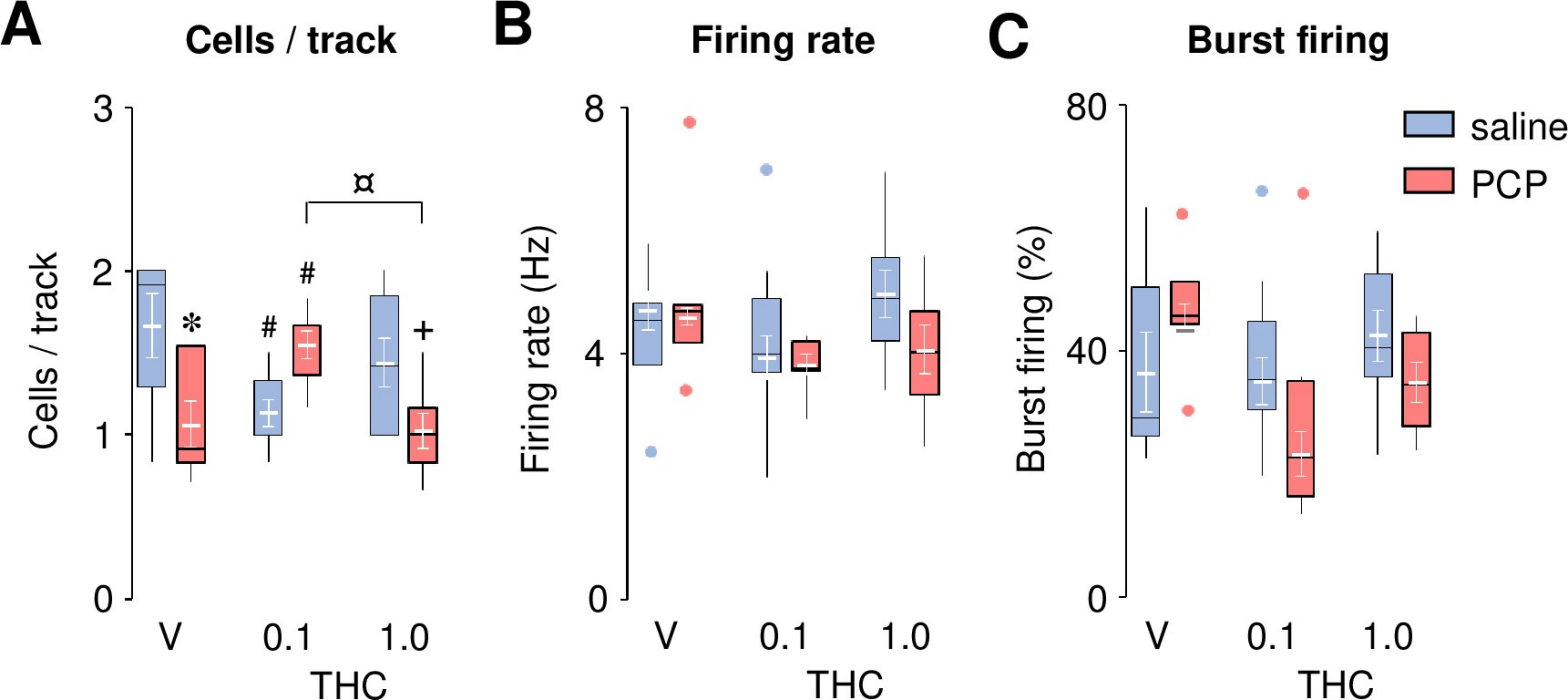
THC reversed PCP-induced decrease in dopamine neuron population activity in the VTA. Effects of THC (0.1 and 1.0 mg/kg) on the number of spontaneously active cells per track (**A**), average firing rate (**B**) and average burst firing (**C**) in saline- and PCP- treated rats. The raw data for this figure are reported in the supplemental file (S1 Raw data) and are summarized here as boxplots computed using Carling’s modification [42]; outliers are depicted as blue (saline) or red (PCP) circles. Values (in white) are expressed as mean ± S.E.M. (n = 6–9 per group). * *P* < 0.05 compared to saline-treated controls, # *P* ≤ 0.05 compared to vehicle (V) controls, + *P* < 0.05 compared to saline-treated vehicle (V) controls, ¤ *P* < 0.05 (Newman-Keuls *post-hoc* test).

As aberrant dopamine function is thought to underlie the positive symptoms of schizophrenia [44], we investigated the effects of THC on the increase of VTA dopamine neuron population activity in PCP-treated rats [39] using an independent set of animals. Surprisingly, PCP-treated rats showed decreased dopamine neuron population activity (Fig 4A). THC, at the lowest dose (0.1 mg/kg), fully reversed this alteration, whereas it significantly reduced the number of spontaneously active cells per track in saline-treated rats. No effect was observed with the highest dose of THC (1.0 mg/kg) in either saline- or PCP-treated animals. ANOVA revealed a trend toward a main effect of Group (*F*_1,37_ = 3.70, *P* = 0.06), no effect of Drug (*F*_2,37_ = 0.61, *P* = 0.55), and a significant interaction between these two factors (*F*_2,37_ = 9.25, *P* < 0.001). This pattern was not accompanied by significant changes in the average firing rate (Fig 4B) nor in the average burst firing (Fig 4C, except for a decrease in burst firing in PCP-treated animals receiving THC 0.1 compared to the saline counterpart). ANOVA for these two variables revealed no effect of Group (*F*_1,33_ = 1.69, *P* = 0.20 and *F*_1,34_ = 0.29, *P* = 0.59, respectively) or interaction (*F*_2,33_ = 0.91, *P* = 0.41 and *F*_2,34_ = 1.00, *P* = 0.38, respectively). There was a Drug effect for the average burst firing (*F*_2,33_ = 3.54, *P* = 0.04), but not for the average firing rate (*F*_2,33_ = 2.94, *P* = 0.07).

The reduction in the number of spontaneously active cells per track was unexpected given a previously published report showing a significant increase in VTA dopamine neuron population activity in PCP-treated rats [39]. However, a closer examination of the data revealed that the number of spontaneously active neurons per track in our saline control animals (1.64 ± 0.17) differed from those published by Aguilar and co-workers (1.05 ± 0.13). In addition, the latter study used Sprague Dawley instead of Wistar rats. To investigate whether difference in rat strain could explain these diverging results, we compared VTA dopamine neuron population activity under baseline conditions in these two strains. In agreement with this hypothesis, Wistar rats showed a higher number of spontaneously active cells per track compared to Sprague Dawley rats (1.63 ± 0.06 versus 0.98 ± 0.08, respectively; *t*_(10)_ = 6.66, *P* < 0.001). No differences in either average firing rate (3.91 ± 0.44 versus 4.19 ± 0.71, respectively; *t*_(10)_ = 0.78, *P* = 0.45) or burst firing (27.87 ± 6.71 versus 35.79 ± 11.88, respectively; *t*_(11)_ = 1.44, *P* = 0.18) were observed.

### Pharmacological targets of THC-mediated effects

As THC can interact with other non-CB_1_ pharmacological targets [46], in an independent set of experiments, we assessed whether the CB_1_ antagonist AM251 (1 mg/kg, i.p.) could reverse the effects of THC (0.1 mg/kg, i.p.) on PCP-induced social withdrawal, PCP-induced elevation of AEA in the nucleus accumbens and PCP-induced decrease in dopamine neuron population activity.

In the EPM task, administration of THC (0.1 mg/kg, i.p.), AM251 (1 mg/kg, i.p.), or both drugs combined had no effect on anxiety-like behavior in either saline- or PCP-treated rats (S2 Fig panels A, B and C).

**Fig 5:**
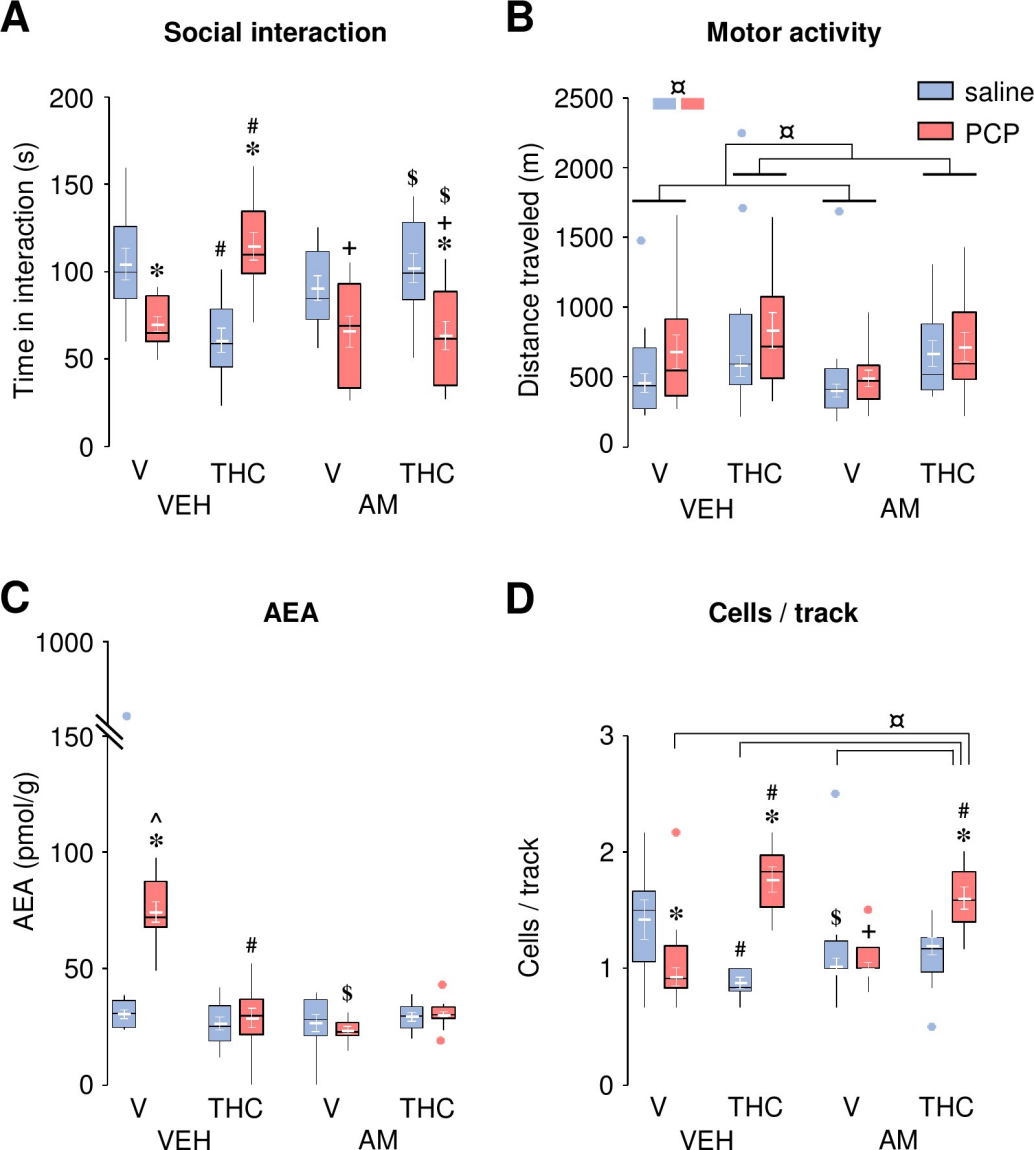
Role of CB_1_ receptors in THC mediated effects. The CB_1_ antagonist AM251 (AM; 1 mg/kg, i.p.) reversed the effects of THC (0.01 mg/kg, i.p.) on social interaction in saline- and PCP-treated animals (**A**). AM251, but not THC, might prevent the trend for increased motor activity after *d*-amphetamine (0.5 mg/kg, i.p.) administration in PCP-treated rats compared to saline-control animals (**B**). Both AM251 and THC blocked PCP-induced increase in AEA levels in the nucleus accumbens (**C**). AM251 failed to reverse THC beneficial effect (in PCP-treated animals) on the number of spontaneously active cells per track in the VTA (**D**). The raw data for this figure are reported in the supplemental file (S1 Raw data) and are summarized here as boxplots computed using Carling’s modification [42];outliers are depicted as blue (saline) or red (PCP) circles. Values (in white) are expressed as mean ± S.E.M. (n = 10–12, 8–12 and 5–8 per group, for the behavioral, neurochemical and electrophysiological data, respectively). * *P* < 0.05 compared to saline-treated controls, # *P* ≤ 0.05 compared to vehicle (V) controls, + *P* < 0.05 compared to saline-treated vehicle (V) controls, $ *P* < 0.05 compared to vehicle (VEH) controls, ^ *P* < 0.05 compared to all other groups (Newman-Keuls *post-hoc* test).

In the social interaction task, THC (0.1 mg/kg, i.p.) reversed PCP-induced social withdrawal, but reduced social interaction in saline-treated rats (Fig 5A), confirming our previous observations. AM251, at a dose (1 mg/kg, i.p.) that did not affect social interaction in controls or social withdrawal in PCP-treated animals [35], fully blocked THC effects in both PCP- and saline-treated rats. ANOVA revealed a main effect of Group (*F*_1,88_ = 3.98, *P* < 0.05), but no effect of Drug (*F*_1,88_ = 0.22, *P* = 0.64), Treatment (*F*_1,88_ = 1.61, *P* = 0.21) or Drug X Treatment interaction (*F*_1,88_ = 0.14, *P* = 0.71); Group X Drug, Group X Treatment, and the three-way interactions were significant (*F*_1,88_ = 11.65, *P* < 0.001, *F*_1,88_ = 14.45, *P* < 0.001, *F*_1,88_ = 21.75, *P* < 0.001, respectively).

During the 30-min habituation period of the motor activity test, THC produced a slight but significant increase in distance traveled in all groups, irrespective of the presence of AM251 (Drug effect: *F*_1,85_ = 13.08, *P* < 0.001, ANOVA did not reveal any other effect: *F*_1,85_ < 1.02, *P* > 0.31; S2 Fig panel D). In presence of *d*-amphetamine (0.5 mg/kg, i.p.), PCP-treated rats showed a trend for enhanced *d*-amphetamine-induced hyperactivity compared to saline-treated controls, irrespective of the presence of THC (Fig 5B left, i.e. VEH pre-treated rats). This difference between saline- and PCP-treated rats was not observed in animals pre-treated with AM251 (Fig 5B right). In addition, THC seemed to increase the distance traveled, similarly to what was already observed during the habituation period. The three-way ANOVA analysis revealed only a main effect of Group (*F*_1,84_ = 5.30, *P* = 0.02; confirming that PCP-treated animals traveled more than saline-treated controls) and Drug (*F*_1,84_ = 8.35, *P* = 0.005, confirming that THC increased the traveled distance compared to vehicle (V)-treated controls), but no other main effect or interactions (*F*_1,84_ < 1.71, *P* > 0.29). Despite the lack of significant interaction, which did not allow us to analyze the data separately for each level of the factor Treatment (VEH and AM251), it appeared that the main effect Group was driven by the VEH (Fig 5B left) but not AM251 (Fig 5B right).

Immediately after the motor activity task, the brains were further processed for endocannabinoid measurements. Confirming our previous observations, THC (0.1 mg/kg, i.p.) fully reversed PCP-induced increase of AEA levels in the nucleus accumbens (Fig 5C). AM251, which alone had no effect, also reversed PCP-induced AEA elevation, suggesting that increased activity at CB_1_ receptors could also contribute to the elevated levels of AEA observed in this experimental group. However, given this confounding outcome, it is not possible to conclude whether AM251 blocked THC effect in PCP-treated rats (i.e. CB_1_-dependend THC effect masked by the same AM251 effect) or not (i.e. CB_1_-independend THC effect). ANOVA revealed a main effect of Group (*F*_1,73_ = 25.94, *P* < 0.001), Drug (*F*_1,73_ = 21.18, *P* < 0.001), Treatment (*F*_1,73_ = 32.99, *P* < 0.001), as well as the following interactions: Group X Drug (*F*_1,73_ = 18.45, *P* < 0.001), Group X Treatment (*F*_1,73_ = 30.78, *P* < 0.001), Drug X Treatment (*F*_1,73_ = 45.22, *P* < 0.001) and three-way interactions (*F*_1,73_ = 27.51, *P* < 0.001). No effect on 2-AG levels were observed in either saline- or PCP-treated animals; irrespective of the presence of THC or AM251 (ANOVA: *F*_1,73_ < 3.04, *P* > 0.08, except for a trend for a significant three-way interaction: *F*_1,73_ = 3.67, *P* = 0.06; S2E Fig).

Finally, we assessed the effects of AM251 on the reversal by THC (0.1 mg/kg, i.p.) of the decreased number of spontaneously active dopamine neurons in PCP-treated rats. As reported above, PCP-treated Wistar rats showed a significant decrease in VTA dopamine neuron population activity that was reversed by THC (Fig 5D). THC also reduced the number of spontaneously active cells per track in saline-treated rats (Fig 5D left). Like THC, AM251 reduced the number of spontaneously active cells per track in saline-treated rats, but did not reverse PCP-induced decrease in dopamine neuron population activity nor THC beneficial effect in PCP-treated animals (Fig 5D right). However, as previously described for AEA levels, it was not possible to conclude whether AM251 blocked the deleterious effect of THC in saline-treated rats (i.e. CB_1_-dependend THC effect masked by the same AM251 effect) or not (i.e. CB_1_-independend THC effect). ANOVA revealed a main effect of Group (*F*_1,48_ = 7.08, *P* = 0.01) and Drug (*F*_1,48_ = 13.27, *P* < 0.001), a significant interaction between these two factors (*F*_1,48_ = 37.85, *P* < 0.001) and a three-way interaction (*F*_1,48_ = 10.15, *P* = 0.003); no effect of Treatment (*F*_1,48_ = 0.38, *P* = 0.54), Group X Treatment (*F*_1,48_ = 0.00, *P* = 0.99) nor Drug X Treatment (*F*_1,48_ = 2.81, *P* = 0.10) interactions were observed. No differences in either average firing rate (*F*_1,50_ < 3.14, *P* > 0.08; S3 Fig panel A) or burst firing (*F*_1,50_ < 2.23, *P* > 0.14; S3 Fig panel B) were observed.

## Discussion

We previously demonstrated that social withdrawal in PCP-treated rats results from deficient endocannabinoid-induced activation of brain CB_1_ receptors [35]. We also showed that this deficit is reversed by endocannabinoid enhancing drugs [33,37]. As endocannabinoids are synthesized and released on-demand after neuronal depolarization [47], they produce a specific spatio-temporal activation of CB_1_ that is not observed with direct agonists, like THC, which cause brain-wide CB_1_ activation. Given this divergence and the negative impact of THC on schizophrenic symptoms reported in the literature [5], we evaluated the effects of THC on PCP-induced social withdrawal and other pre-clinical correlates of schizophrenia. Our data showed that THC at the lowest (0.1 mg/kg), but not at the highest dose (1 mg/kg), reversed PCP-induced effects, including 1) social withdrawal, 2) elevation of AEA in the nucleus accumbens and 3) VTA dopamine neuron population decreased activity. However, in control rats, THC dose-dependently produced deficits similar to those observed in PCP-treated animals (social withdrawal and aberrant dopamine activity).

Cannabis is usually consumed for recreational purposes (i.e., pleasure-seeking, relaxation or the so-called ‘high’) by the general population [36] as well as by psychotic patients [48]. In animal studies, THC produces aversive effects if administered at high doses (1–300 mg/kg) [49] and characteristic physiological and behavioral alterations known as the “tetrad” (ED_50_ for hypolocomotion: 69 mg/kg, hypothermia: 44 mg/kg, catalepsy: 18 mg/kg, and analgesia: 12 mg/kg [50]). THC administration may not entirely mimic the effects of smoking cannabis, which contains more than 104 different cannabinoids including cannabidiol. Also, different effects may result from diverse cannabis preparations, which vary in cannabinoid content and potency [51]. Furthermore, THC route of administration can significantly affect its pharmacokinetic, pharmacodynamic and behavioral effects, making it difficult to identify a Human Equivalent Dose (HED). THC dosing is however critical to ascertain its effect on a specific behavior given the biphasic nature of its pharmacological action, a property shared with other exogenous cannabinoids [52]. For instance, acute administration of low doses of cannabinoids induces anxiolytic-like responses in rodents, whereas higher doses produce anxiety-like reactions [52]. In rats, an anxiolytic-like effect of THC in the EPM was reported with doses between 0.075 and 1.5 mg/kg [53]. When using a similar dose range, we did not see a comparable effect in our study probably because of different baseline levels of anxiety-like behavior (%T_open_) in control animals (%T_open_ = 36% in our study vs. 7% in the Rubino and co-workers’s paper; estimated from Fig 2), suggesting that our experimental conditions are more prone to detect anxiogenic- rather than anxiolytic-like responses. As recreational users often self-administer cannabis to achieve a state of relaxation, one could infer that THC would be used in experimental models at doses producing an anxiolytic-like reaction. However, THC is often administered in pre-clinical [54] or even clinical settings [55–57] at high, anxiety-like producing doses, despite these studies claim to use doses relevant for human consumption. In our study, we selected doses that were rewarding to Wistar rats (0.1 and 0.3 mg/kg), as indicated by their ability to induce place preference [58], and within the range reported by Rubino et al. [53] to produce anxiolytic-like effects. After excluding confounding effects on motor activity (see Material and Method), we found that THC administration at the lowest (0.1 mg/kg), but not the highest (1 mg/kg; a dose that did not produce place preference in Braida’s study [58]), dose had beneficial effects on various correlates of schizophrenia (see above). While there is a large literature on THC exposure in adolescent rodents, few studies [59–64] have assessed THC effects on schizophrenia-related behavioral deficits in adult rodents. For instance, Malone et al., [62] reported that the prepulse inhibition deficit (an operational measure of sensorimotor gating) observed in adult rats reared in isolation was worsened by THC (1 and 3 mg/kg, i.v.). Rodriguez et al [63] showed that THC (5 mg/kg, i.p.) exacerbated working memory deficits induced by neonatal exposure to PCP in mice. Although our data obtained with the highest dose of THC (1 mg/kg) agree with the studies reported above, to our knowledge we are the first to show that a low dose of THC (0.1 mg/kg) can improve social withdrawal and other pre-clinical correlates of schizophrenia.

PCP-induced social withdrawal, which results from a deficient stimulation of brain cannabinoid CB_1_ receptors [35], is also reversed in a CB_1_-dependent manner by systemic administration of the AEA-enhancing drug URB597 [35]. Similar observations were reported with JZL184, an inhibitor of the catabolic enzyme of 2-AG [37], the cannabinoid transporter inhibitor OMDM-2 [33], the cannabinoid agonist CP55,940 (at the very low dose of 0.01 mg/kg) [35], and following self-administration (dosage was titrated by each animal) of the cannabinoid agonist WIN55,212–2 [34]. The behavioral amelioration observed with these drugs might result from the restoration of CB_1_-mediated inhibition of GABAergic neurons in the amygdala [35]. Indeed, although endocannabinoids can regulate both excitatory and inhibitory inputs, GABAergic synapses are more sensitive to endocannabinoid-mediated effects [47], possibly because of the higher expression of CB_1_ receptors in inhibitory versus excitatory afferents [65]. This anatomical specificity is lost when exogenous cannabinoids are applied at high doses or when the endocannabinoid tone is enhanced above physiological levels [66]. In keeping with this hypothesis, we previously showed that blockade of AEA degradation in saline-control animals, leading to elevated AEA brain levels, caused social withdrawal [35]. On the other hand, endocannabinoid elevation in PCP-treated animals, which showed deficient AEA mobilization [35], reversed their social behavior deficit. Thus, our data on the effect of THC in PCP-treated animals could be interpreted through a similar mechanism of action. Specifically, in control animals, THC (even at the lowest dose) would add onto the existing endocannabinoid-mediated CB_1_ stimulation, recruiting additional CB_1_ receptors expressed on glutamatergic terminals and leading to social withdrawal. On the other hand, the CB_1_ antagonist AM251 (1 mg/kg) would restore a normal social interaction in these animals by producing a rightward shift of the THC dose-response curve. In PCP-treated rats, given a pre-existing lower endocannabinoid levels [35], THC would preferentially target CB_1_ receptors on GABAergic neurons only at the lowest dose (0.1 mg/kg), but it would also recruit CB_1_ receptors expressed on glutamatergic terminal at higher doses (1 mg/kg).

Previous clinical studies have reported higher levels of AEA in the blood and CSF of patients with acute schizophrenia [24,43,67]. Interestingly, we consistently observed AEA elevation in the nucleus accumbens of PCP-treated animals either in resting condition [33], or after engaging in social interaction [35], or after *d*-amphetamine administration (present study). Whereas the mechanism responsible for AEA elevation in schizophrenia is still a matter of debate, activation of dopamine D_2_ receptors has been shown to enhance AEA concentrations in rat brain [68,69], indicating that this elevation might result from increased dopaminergic activity [24]. Alternatively, it has been proposed that only stimulation of D_2_ auto-receptor, which decreases dopamine release and consequently its inhibitory control over cortico-striatal glutamatergic transmission, can increase AEA [69]. According to this view, the AEA elevation reported in schizophrenic patients might not be driven by increased dopaminergic activity but rather by increased glutamatergic transmission, likely arising from unbalanced excitation/inhibition within specific microcircuitries [70]. In agreement with this scenario, we previously reported that accumbal AEA elevation in PCP-treated animals under resting conditions is not accompanied by changes in dopamine levels [33]. In this study, in which AEA measurements were carried out after *d*-amphetamine administration, PCP-treated animals did not show enhanced *d*-amphetamine-induced hyperactivity, a behavioral phenotype generally associated with mesolimbic dopaminergic hyper-responsivity [71]. Yet, these animals showed increased AEA levels. Interestingly, while the first hypothesis suggested that AEA production may result from increased dopaminergic activity [24], the latter postulates that increased dopaminergic activity, by negatively modulating glutamatergic transmission, would instead dampen AEA mobilization [69]. Accordingly, THC administration, which increases dopamine transmission in the nucleus accumbens [72], should lead to either an increase in AEA production according to the first hypothesis [68], or to a reduction in AEA levels according to the second one [69]. We showed that THC reversed PCP-induced accumbal AEA elevation without affecting endocannabinoid levels in control animals. Surprisingly, the CB_1_ antagonist AM251, like THC, also reversed PCP-induced accumbal AEA elevation. A possible explanation of these results is that THC, given its low efficacy at CB_1_ receptors [46], might behave as an antagonist if the receptor reserve is limited, as in the case of glutamatergic terminals [73]. In keeping with this idea, cannabinoid agonists and antagonists produced similar outcomes in our electrophysiological experiments, and several behavioral paradigms: EPM [74], working memory [33] and social novelty preference [75]. Our data are also consistent with a report showing that the elevation of AEA in drug-naïve schizophrenics is dramatically reduced in patients consuming cannabis frequently but not in healthy individuals [26]. Finally, we observed a THC-induced decrease in the phosphorylated form of GSK3β (inactive state), which could be indicative of increased dopamine transmission [45]. Of note, reduced phosphorylation of GSK3β at Ser9 was described in schizophrenic individuals [76] and a recent study revealed an association between an AKT polymorphism and increased psychotomimetic symptoms after smoking cannabis [77].

As previously mentioned, excessive stimulation of striatal dopamine D_2_ receptors are presumably responsible for positive symptoms, whereas deficient stimulation of prefrontal dopamine D_1_ receptors has been implicated in cognitive impairment and negative symptoms [44]. Yet, experimental evidence indicates that striatal hyper-dopaminergia does not occur in all patients [78]. Since heightened sensitivity to *d*-amphetamine in schizophrenics has been suggested to reflect aberrant mesolimbic dopamine transmission, *d*-amphetamine-induced hyperactivity is commonly utilized to model the positive symptoms in rodents, even though this approach has been recently challenged [79]. A more direct way to assess dopaminergic alterations is by measuring the number of spontaneously active VTA dopamine neurons, which is highly correlated with tonic levels of extrasynaptic dopamine in the nucleus accumbens [80]. Indeed, a greater number of spontaneously active VTA dopamine neurons was observed in the methylazoxymethanol acetate (MAM) rat model of schizophrenia [81], as well as in the sub-chronic PCP rat model [39]. Surprisingly, in our study, PCP-treated rats showed a lower number of spontaneously active VTA dopaminergic neurons when compared to controls. This unexpected outcome was due to the different rat strain used (Wistar vs. Sprague Dawley). Several differences between these two strains have been described in earlier reports with respect to the effects of dopamine receptor agonists on prepulse inhibition [82], and in the context of modeling schizophrenia [83,84]. For example, in addition to being less anxious [85] and more sensitive to NMDA receptors antagonists [82], Sprague Dawley rats exhibit reduced locomotor activity compared to Wistar rats [83]. These differences, which could be related–among other things [85]–to a different expression of dopamine receptors [86], may also result from a lower number of spontaneously active dopamine neurons (as observed in our study). As population activity may regulate the responsivity of the dopaminergic system [80], it might also explain the divergent behavioral response to *d*-amphetamine in Sprague Dawley vs. Wistar rats [33,71]. Indeed, while the formers have a low number of spontaneous active dopaminergic neurons that can be further activated, Wistar rats show a sub-maximal activation leading to a possible ceiling effect. It was recently proposed that this sub-maximal activation would be expected to greatly lessen the amplitude of the dopamine response to stimuli and therefore to decrease the rewarding value of external stimuli [87]. In addition, social interaction has been shown to be driven by the activation of VTA-to-nucleus accumbens projections [88]. In agreement with our electrophysiological data and the observation that PCP-treated animals show a deficit in conditioned place preference for social contact [89], we postulated that PCP-induced social withdrawal results from social anhedonia [41,75]. Thus, by increasing dopaminergic transmission in the nucleus accumbens [72], THC could improve social withdrawal in PCP-treated animals and reverse the PCP-induced aberrant decrease in dopamine neuron population activity. Interestingly, a recent imaging study showed that the hypo-connectivity found in schizophrenic patients between the nucleus accumbens and the prefrontal cortical regions involved in reward processing (such as the orbitofrontal cortex) was improved by both cannabis and THC administration [90]. In addition, even though the role of the orbitofrontal cortex in regulating mesolimbic dopamine transmission is not well documented, it has been suggested that it might strongly affect the spontaneous activity of VTA neurons [91]. In agreement with this hypothesis, we previously showed that a deficit in orbitofrontal cortex neuronal activation induced by social interaction in PCP-treated animals can be reversed by cannabinoids [92].

A large body of literature has consistently linked cannabis use to the exacerbation of positive symptoms of schizophrenia. So far, very few studies have investigated whether THC exacerbates schizophrenia-like symptoms in adult animals [59–64]. Our study shows that THC can produce a broad range of deficits in healthy animals that resembled those observed in animal models of schizophrenia. However, it also shows that THC can improve some behavioral deficits associated with schizophrenia only if used at low doses known to be rewarding [58], anxiolytic-like [53], and comparable to those used by humans to seek pleasure or achieve a state of relaxation. Our data may reconcile several controversial findings and provide a new framework supporting the self-medication hypothesis of cannabis use to alleviate negative symptoms. Finally, given the growing use of marijuana for medical and recreational purposes, and the dramatic increase in potency of cannabis preparations over the years [51], our results indicate that THC dosing has important implications for future preclinical and clinical research, as well as policy making.

## Material and methods

### Subjects

Male Wistar rats (200–225 g; Charles River Laboratories, Wilmington, MA) were housed 2 per cage at 22 ± 1°C, under a 12 h light-dark cycle with food and water available *ad libitum*, and habituated to the housing conditions for one week prior to the experiments. Animals were treated sub-chronically (twice a day for 7 days, at approximately 8:00 a.m. and 8:00 p.m.) with either saline (1 ml/kg) or PCP (5 mg/kg) via intraperitoneal route (i.p.) and tested starting 5 days after the last drug injection; no PCP was on board or administered during the behavioral assessment and electrophysiological recordings. Therefore, given an approximate age of 49–52 days upon arrival, the animals were young adults at the time of experimentation. All experiments were carried out in accordance with the National Institute of Health Guide for the Care and Use of Laboratory Animals and approved by the Institutional Animal Care and Use Committee of the University of Texas Health Science Center at San Antonio.

For the experiment “Wistar vs. Sprague Dawley rats”, male Wistar rats (Charles River Laboratories, Wilmington, MA) were compared to male adult Sprague Dawley rats (Harlan Laboratories, Indianapolis, IN).

### Behavioral assessment

All experimental procedures were carried out in the morning (during the light portion of the cycle) in a room adjacent to the vivarium. Specifically, two independent experiments were conducted, each with a new group of rats. In the first experiment (Fig 1), 112 animals (14 per group) were tested for anxiety, using the elevated plus maze (EPM) task, social interaction and motor activity, 5, 7 and 8/9 days after the last drug injection, respectively (behavioral tests are described below). In the second experiment (Fig 5), 96 animals (12 per group) were tested using the same behavioral battery. The order of execution of the behavioral tests was: EPM task, social interaction test, and motor activity in response to *d*-amphetamine.

#### Elevated plus maze

Anxiety-like behaviors were assessed using the EPM task, five days after the last drug injection, as previously described [74], with the only exception that the EPM was located in a well-lit room. Data were collected, by an experimenter blind to the study, as total number of entries (TE = E_open_ + E_closed_ + E_center_) and time spent in each arm and/or center of the EMP (the EPM center was coded as neither open nor closed arms). An entry was defined as a rat entering the arm with all four paws. Levels of anxiety were assessed as percent time spent in the open arms [%T_open_ = T_open_ / (T_open_ + T_closed_)] and percent of open arm entries [%E_open_ = E_open_ / (E_open_ + E_closed_)].

#### Social interaction

Social interaction was assessed using the dyadic paradigm, as previously described [41]. Six days after the last drug injection, animals were familiarized to an arena made of black acrylic (100 cm x 100 cm x 40 cm) and located in a dimly lit room (5 lux at the arena center). Rats were placed individually in the center of the arena and free exploration was allowed for 30 min. On the following day, animals were tested in pairs (two unfamiliar rats receiving the same treatment, and housed in different home cages) and matched up according to their body weights. Animals were placed simultaneously into the arena and their behavior recorded by a video camera for 10 min. An experimenter blind to the study scored the total time spent by each rat actively engaging in the following social behaviors: (1) investigative sniffing (sniffing the conspecific's snout or other body parts of the body including the anogenital region); (2) following (moving towards and following the conspecific around the arena); (3) climbing over or under (climbing over the conspecific's back or pushing the head and forepart of the body under the conspecific).

#### Motor activity

Eight or nine days after the last drug injection, rats were placed in Actimot boxes (TSE Systems GmbH, Bad Homburg, Germany; located in a brightly lit room) for motor activity assessment. After a 30-min habituation period, they received a *d*-amphetamine injection (0.5 mg/kg, i.p.) and motor activity was recorded for an additional 90-min period. Distance traveled was measured using the ActiMot Activity Measuring System version 6.07 (TSE Systems GmbH, Bad Homburg, Germany).

### Neurochemical and molecular studies

#### Tissue sampling

Immediately after the motor activity test (see above), animals were anesthetized with halothane, their brains rapidly collected, frozen in 2-methylbutane (- 45°C), and stored at—80°C until use. Frozen brains were placed on a stainless steel mould (Roboz; Rockville, MD) kept at—17°C and sliced into 1-mm coronal sections using razor blades to dissect out the nucleus accumbens–a brain area for which we reported AEA elevation in PCP-treated animals independently of the physiological state of the animal, that is irrespectively of the behavioral task involved or lack thereof [33,35]. In particular, this elevation contrasts with the deficient endocannabinoid mobilization observed in the amygdala and prefrontal cortex of the same PCP-treated rats during social interaction [35].

#### Gas chromatography / mass spectrometry

The endocannabinoids AEA and 2-AG were quantified by gas chromatography/mass spectrometry (GC/MS) as previously described [33], using an isotope dilution assay [93]. Tissue samples were spiked with 50 pmol of [^2^H_4_]AEA and [^2^H_5_]-2-AG (internal standards) and lipids were extracted by adding methanol/chloroform/water (1 : 2 : 1, v/v/v). The chloroform layer was further purified by solid phase extraction using C18 Bond Elut cartridges (100 mg; Varian, USA). Endocannabinoid-containing fractions were processed, derivatized and analyzed by GC/MS as previously described [93].

#### Western blot

The activity of the Akt/GSK3β signaling pathway was assessed by Western blot, following a procedure previously described [35]. Briefly, tissue samples (nucleus accumbens) were homogenized in ice-cold lysis buffer, centrifuged at 14000 rpm for 20 min and supernatants collected for further analysis. Equal amounts of protein (20 μg) were resolved by 10% SDS-PAGE and transferred to a PVDF membrane (0.2 μm). Membranes were incubated in 3% non-fat milk in TBS-T for 1 h followed by overnight incubation in primary antibody [anti-Akt (1:1000; Cell Signaling), anti-phosphorylated Akt (pAkt at Thr 308; 1:500; Cell Signaling), anti-GSK3β (1:1000; Cell Signaling), anti-phosphorylated GSK3β (pGSK3β at Ser 9; 1:500; Cell Signaling), and anti-β-Actin (1:10000; Sigma Chemical)] at 4°C. Membranes were then washed with TBS-T followed by incubation with an appropriate HRP-conjugated secondary antibody (1:2000; Santa Cruz) for 60 min at room temperature. Protein bands were visualized using the ECL kit (Amersham, GE Healthcare, Buckinghamshire, England) followed by exposure to X ray. Band immunoreactivity was quantified by densitometry using the NIH image software.

### *In vivo* electrophysiology

Dopamine neuron population activity in the VTA was measured between fourteen and fifty one days after the last drug injection, as previously described [39]. Briefly, rats were anesthetized with 8% chloral hydrate (400 mg/kg, i.p.), as this anesthetic does not significantly depress dopamine neuron activity [94], and placed in a stereotaxic apparatus where body temperature was maintained at 37°C by a thermostatically controlled heating pad. Anesthesia was maintained by supplemental administration of chloral hydrate as required to maintain suppression of limb-compression withdrawal reflex. The use of this anesthetic for dopamine neuron recordings was approved by our Institutional Animal Care and Use Committee. Glass micro-electrodes (impedance 6–14 MΩ) were lowered into the VTA (Bregma: A -5.3 mm, L +0.6 mm, V– 6.5 to -9.0 mm). Population activity (defined as the number of spontaneously-active dopamine cells per vertical electrode track), basal firing rate, and the proportion of action potentials occurring in bursts were recorded. Between 5 and 7 tracks (separated by 0.2 mm) were completed for each animal. Representative traces from VTA dopamine recordings are shown in S4 Fig.

Three independent experiments were conducted, each with a new group of rats. In the first experiment (Fig 4), the effects of THC (0.1 and 1.0 mg/kg) were tested in 54 animals (9 per group). In the second experiment (“Wistar vs. Sprague Dawley rats”), we used 14 rats (7 per group). In the third experiment (Fig 5D), we assessed whether the CB_1_ antagonist AM251 (1 mg/kg, i.p.) could reverse the effects of THC (0.1 mg/kg, i.p.) in 72 rats (9 per group).

### Drugs

PCP and *d*-amphetamine were purchased from Sigma/RBI (St Louis, MO), AM251 was from Cayman Chemical (Ann Arbor, MI) and THC (in ampoule; 200 mg/ml in absolute ethanol) was obtained from the National Institute for Drug Abuse. Drugs were prepared freshly and injected i.p.. The dose range for THC was first based on a conditioned place preference study [58] and an EPM study [53] (see discussion). Specifically, Braida et al. [58] assessed the effects of a wide range of doses of THC–form very low to moderate (0.015–6 mg/kg, i.p.)–on conditioned place preference, and showed that THC induced reward in Wistar rats, only at the lowest doses (0.075–0.75 mg/kg); the highest dose (6 mg/kg) producing place aversion (58). Similarly, Rubino et al., [53] investigated the effect of low doses of THC (0.015–3 mg/kg) on anxiety-like behavior in rats and reported anxiolytic effects within a similar dose range (0.075–1.5 mg/kg). In addition, preliminary experiments were conducted to determine doses of THC that would not interfere with the motor skills of the animals, a potential confounder for the EPM and social interaction tasks. Specifically, dose–response curves (THC; 0.1, 0.3, 1 and 3 mg/kg, i.p) were established for horizontal (S5 Fig panel A) and vertical (S5 Fig panel B) motor activity in control animals; the 3 mg/kg dose was discarded as it reduced the number of rearing. Thus, in this study, animals received an acute injection of either vehicle (Tween80:PEG:saline, 5:5:90, 1 ml/kg, i.p.) or THC (0.1, 0.3 or 1 mg/kg, i.p.) 30 min before the behavioral assessment or THC (0.1 or 1 mg/kg, i.p.) 10 min before the electrophysiological recording. To investigate the pharmacological mechanisms underlying THC effects, an independent cohort of rats were pre-treated with the selective CB_1_ antagonist AM251 (1 mg/kg, i.p.) or its vehicle (Tween80:PEG:saline, 10:10:80, 1 ml/kg, i.p.) immediately before THC (0.1 mg/kg, i.p.). The dose of AM251 was chosen from previous *in vivo* studies [35,95].

### Statistical analysis

Outliers were filtered by Carling’s method, namely the median rule, using the recommended ideal fourths and the constant k_2_ adjusted for the sample size [k_2_ = (17.63n – 23.64)/(7.74n – 3.71)] [42]. Data were analyzed by two-way ANOVA with Group (saline, PCP) and Drug (THC; 4 or 3 levels) as between-subject factors. For the pharmacological studies (pre-treatment with AM251), data were analyzed by three-way ANOVA with Group (saline, PCP), Drug (vehicle, THC) and Treatment (vehicle, AM251) as between factors. When required, multiple comparisons were performed using the Newman-Keuls test with the level of significance set at p < 0.05. For comparison of two independent groups (Wistar vs. Sprague Dawley rats), unpaired two-tailed Student’s t-test was used.

## Supporting information

S1 Fig Effects of THC (0.1–1.0 mg/kg) on motor activity in saline- and PCP- treated rats. Distance traveled in the Actimot activity box in a novel environment (i.e. during the 30-min habituation period). The raw data for this figure are reported in the supplemental file (S1 Raw data) and are summarized here as boxplots computed using Carling’s modification (Carling, 2000); outliers are depicted as blue (saline) circles. Values (in white) are expressed as mean ± S.E.M. (n = 13–14 per group). ANOVA did not reveal any main effect of Group (*F*_1,103_ = 0.74, *P* = 0.39), Drug (*F*_3,103_ = 1.35, *P* = 0.26), or interaction between these two factors (*F*_3,103_ = 1.23, *P* = 0.30). V, vehicle.
(TIF)

S2 Fig Role of CB_1_ receptors in THC mediated effects in saline- and PCP- treated rats. Percent time spent in the open arms (%T_open_; **A**), percent of open arms entries (%E_open_; **B**) and total number of entries (TE; **C**) in the EPM task. Distance traveled in the Actimot activity box in a novel environment (**D**). 2-AG levels in the nucleus accumbens (**E**). The raw data for this figure are reported in the supplemental file (S1 Raw data) and are summarized here as boxplots computed using Carling’s modification (Carling, 2000); outliers are depicted as blue (saline) or red (PCP) circles. Values (in white) are expressed as mean ± S.E.M. (n = 8–12 per group). ANOVA revealed no effect or interaction for %T_open_ (*F*_1,88_ < 1.97, *P* > 0.16), %E_open_ (*F*_1,88_ < 3.06, *P* > 0.08) and 2-AG levels (*F*_1,73_ < 3.04, *P* > 0.08). For TE, ANOVA revealed a main effect of Group (*F*_1,86_ = 8.14, *P* < 0.01) and Treatment (*F*_1,86_ = 5.12, *P* < 0.05), but no effect of Drug (*F*_1,86_ = 0.93, *P* = 0.34), or any interaction (*F*_1,86_ < 2.69, *P* > 0.10). For the motor activity, revealed a main effect of Drug (*F*_1,85_ = 13.08, *P* < 0.001), but no other effect or interaction (*F*_1,85_ < 1.01, *P* > 0.31). ¤ *P* < 0.05 (Newman-Keuls *post-hoc* test). AM, AM251 (1 mg/kg); T, THC (0.1 mg/kg); V and VEH, vehicle.
(TIF)

S3 Fig Average firing rate burst firing in the VTA. Absence of effects of THC (T; 0.1 mg/kg) and/or AM251 (AM; 1 mg/kg) on average firing rate (**A**) and average burst firing (**B**) in saline- and PCP- treated rats. The raw data for this figure are reported in the supplemental file (S1 Raw data) and are summarized here as boxplots computed using Carling’s modification (Carling, 2000); outliers are depicted as blue (saline) or red (PCP) circles. Values (in white) are expressed as mean ± S.E.M. (n = 5–8 per group). ANOVA revealed no effect or interaction for average firing rate (*F*_1,50_ < 3.14, *P* > 0.08) and average burst firing (*F*_1,50_ < 2.23, *P* > 0.14). V and VEH, vehicle.
(TIF)

S4 Fig Representative trace and action potential from VTA dopamine neuron recordings. Electrophysiological traces, as well as dopamine neurons waveforms (inserts), are shown for saline- (**A**) and PCP- (**B**) treated animals.
(TIF)

S5 Fig Effects of THC on horizontal and vertical motor activity. Distance traveled (**A**) and number of rearing (**B**) in the Actimot activity box in a novel environment following THC (0.3–3 mg/kg, i.p.) administration. The raw data for this figure are reported in the supplemental file (S1 Raw data) and are summarized here as boxplots computed using Carling’s modification (Carling, 2000); outliers are depicted as gray circles. Values (in white) are expressed as mean ± S.E.M. (n = 6–8 per group). ANOVA revealed no effect for horizontal (*F*_3,25_ = 0.65, *P* = 0.59), but one for vertical activity (*F*_3,25_ = 7.69, *P* < 0.001). * *P* < 0.001 compared to vehicle (V) control, ¤ *P* < 0.01 (Newman-Keuls *post-hoc* test).
(TIF)

S1 Raw images (PDF)

S1 Raw data (XLSX)

## Abbreviations

2-AG: 2-arachidonyl glycerol
AEA: anandamide
EPM: Elevated Plus Maze
GC/MS: gas chromatography/mass spectrometry
PCP: phencyclidine
THC: Δ9-tetrahydrocannabinol
VTA: ventral tegmental area
