# Supplementary figures and images for: Differential effects of Δ9-tetrahydrocannabinol dosing on correlates of schizophrenia in the sub-chronic PCP rat model
Source: PLoS One. 2020 Mar 12;15(3):e0230238. doi: 10.1371/journal.pone.0230238 (PMC7067407; doi:10.1371/journal.pone.0230238)

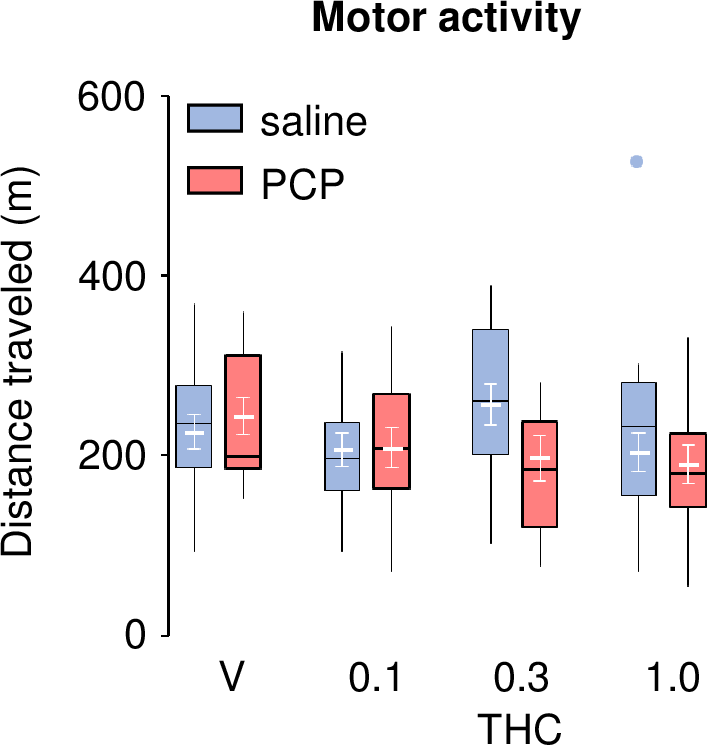

Supplement: S1 Fig — Distance traveled in the Actimot activity box in a novel environment (i.e. during the 30-min habituation period). The raw data for this figure are reported in the supplemental file (S1 Raw data) and are summarized here as boxplots computed using Carling’s modification (Carling, 2000); outliers are depicted as blue (saline) circles. Values (in white) are expressed as mean ± S.E.M. (n = 13–14 per group). ANOVA did not reveal any main effect of Group (F1,103 = 0.74, P = 0.39), Drug (F3,103 = 1.35, P = 0.26), or interaction between these two factors (F3,103 = 1.23, P = 0.30). V, vehicle. (TIF) [file pone.0230238.s001.tif]

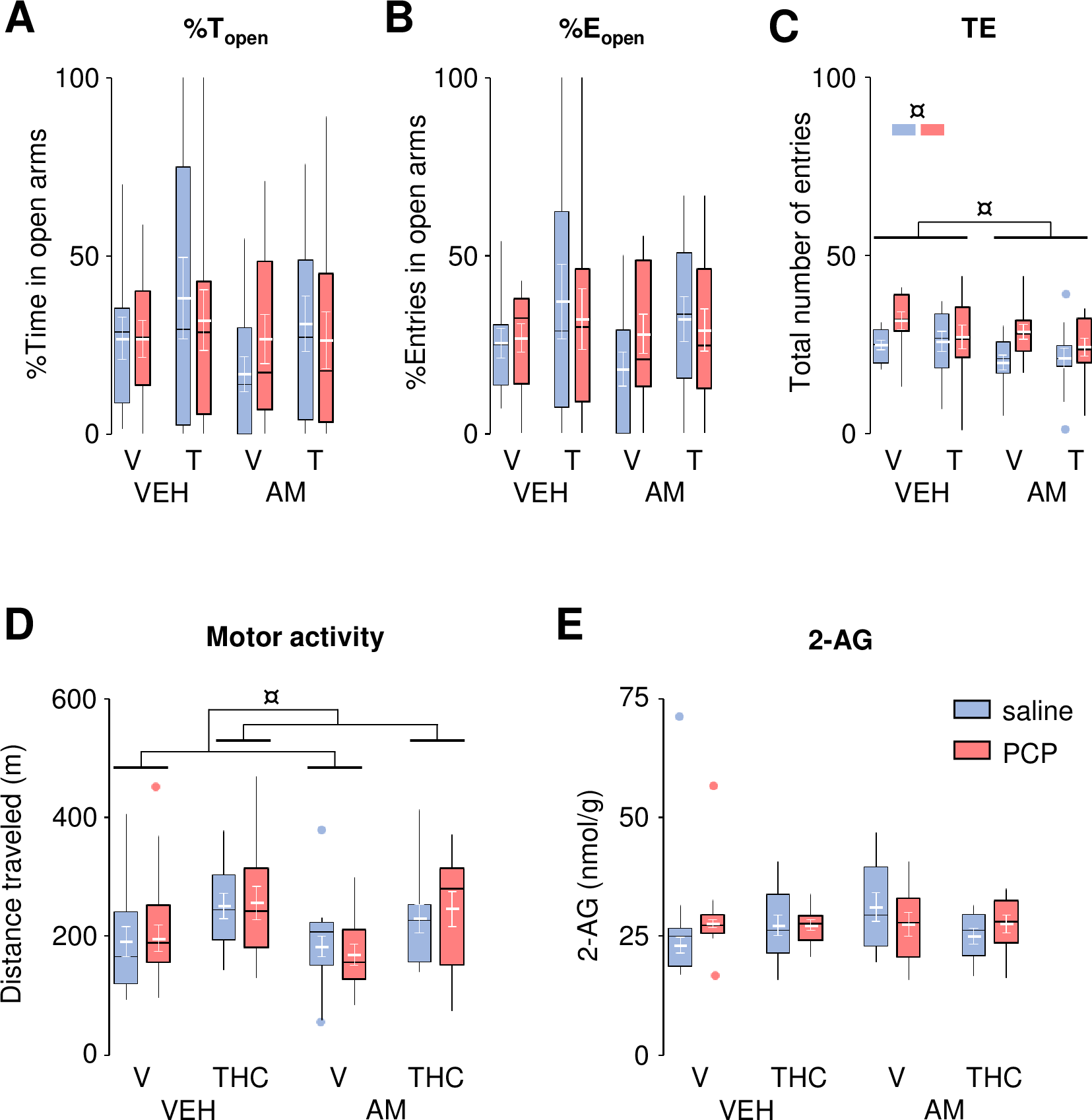

Supplement: S2 Fig — Percent time spent in the open arms (%Topen; A), percent of open arms entries (%Eopen; B) and total number of entries (TE; C) in the EPM task. Distance traveled in the Actimot activity box in a novel environment (D). 2-AG levels in the nucleus accumbens (E). The raw data for this figure are reported in the supplemental file (S1 Raw data) and are summarized here as boxplots computed using Carling’s modification (Carling, 2000); outliers are depicted as blue (saline) or red (PCP) circles. Values (in white) are expressed as mean ± S.E.M. (n = 8–12 per group). ANOVA revealed no effect or interaction for %Topen (F1,88 < 1.97, P > 0.16), %Eopen (F1,88 < 3.06, P > 0.08) and 2-AG levels (F1,73 < 3.04, P > 0.08). For TE, ANOVA revealed a main effect of Group (F1,86 = 8.14, P < 0.01) and Treatment (F1,86 = 5.12, P < 0.05), but no effect of Drug (F1,86 = 0.93, P = 0.34), or any interaction (F1,86 < 2.69, P > 0.10). For the motor activity, revealed a main effect of Drug (F1,85 = 13.08, P < 0.001), but no other effect or interaction (F1,85 < 1.01, P > 0.31). ¤ P < 0.05 (Newman-Keuls post-hoc test). AM, AM251 (1 mg/kg); T, THC (0.1 mg/kg); V and VEH, vehicle. (TIF) [file pone.0230238.s002.tif]

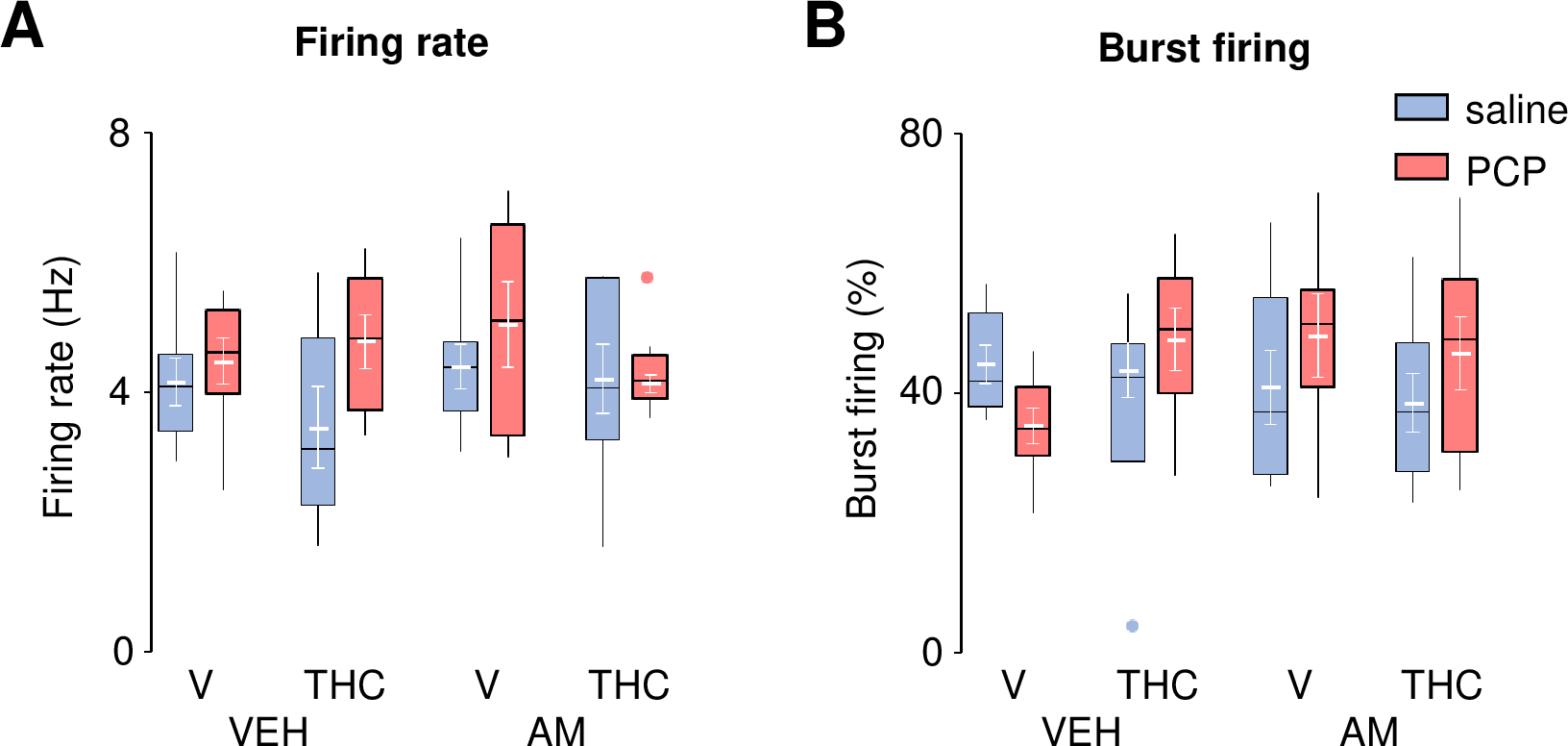

Supplement: S3 Fig — Absence of effects of THC (T; 0.1 mg/kg) and/or AM251 (AM; 1 mg/kg) on average firing rate (A) and average burst firing (B) in saline- and PCP- treated rats. The raw data for this figure are reported in the supplemental file (S1 Raw data) and are summarized here as boxplots computed using Carling’s modification (Carling, 2000); outliers are depicted as blue (saline) or red (PCP) circles. Values (in white) are expressed as mean ± S.E.M. (n = 5–8 per group). ANOVA revealed no effect or interaction for average firing rate (F1,50 < 3.14, P > 0.08) and average burst firing (F1,50 < 2.23, P > 0.14). V and VEH, vehicle. (TIF) [file pone.0230238.s003.tif]

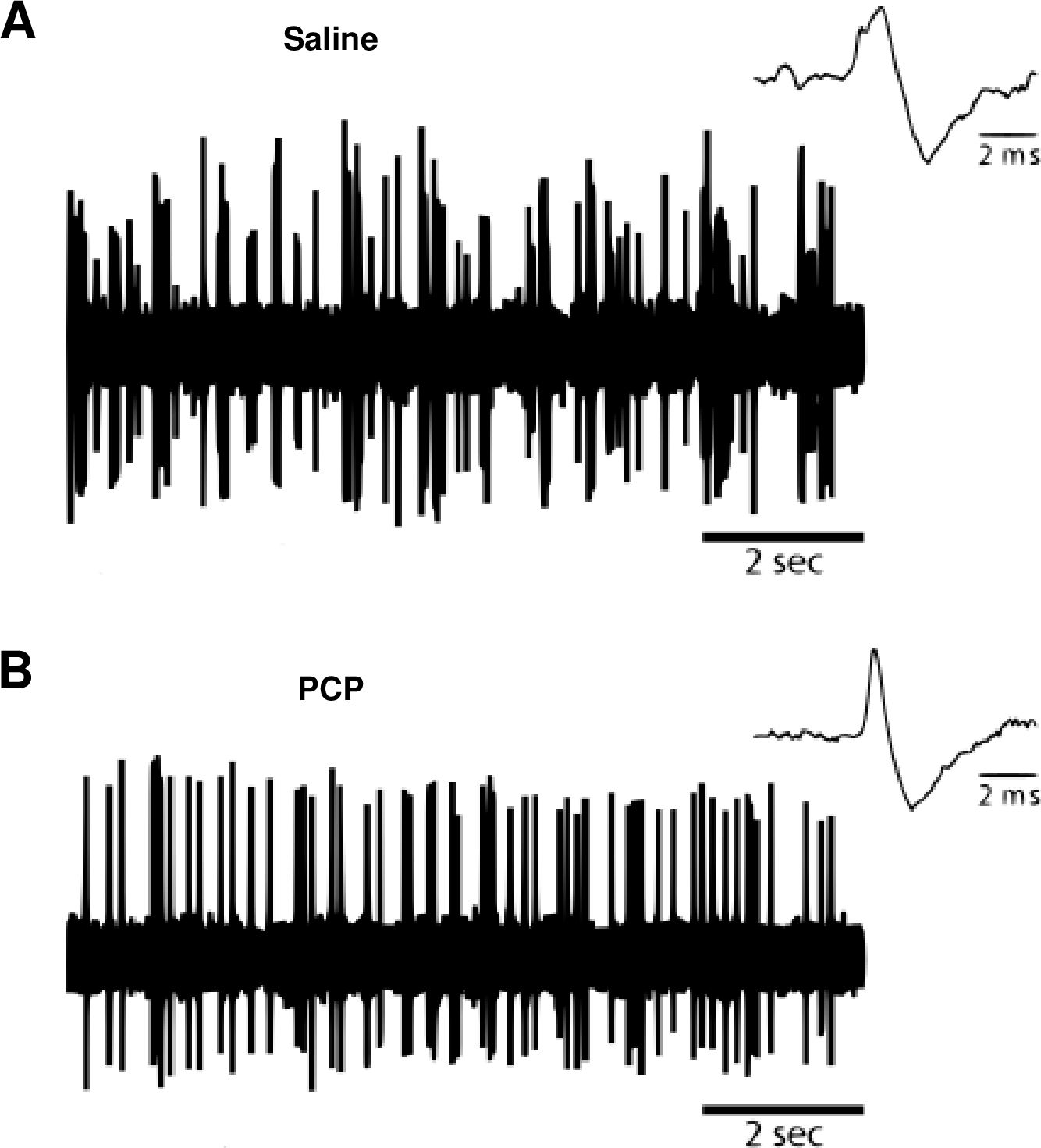

Supplement: S4 Fig — Electrophysiological traces, as well as dopamine neurons waveforms (inserts), are shown for saline- (A) and PCP- (B) treated animals. (TIF) [file pone.0230238.s004.tif]

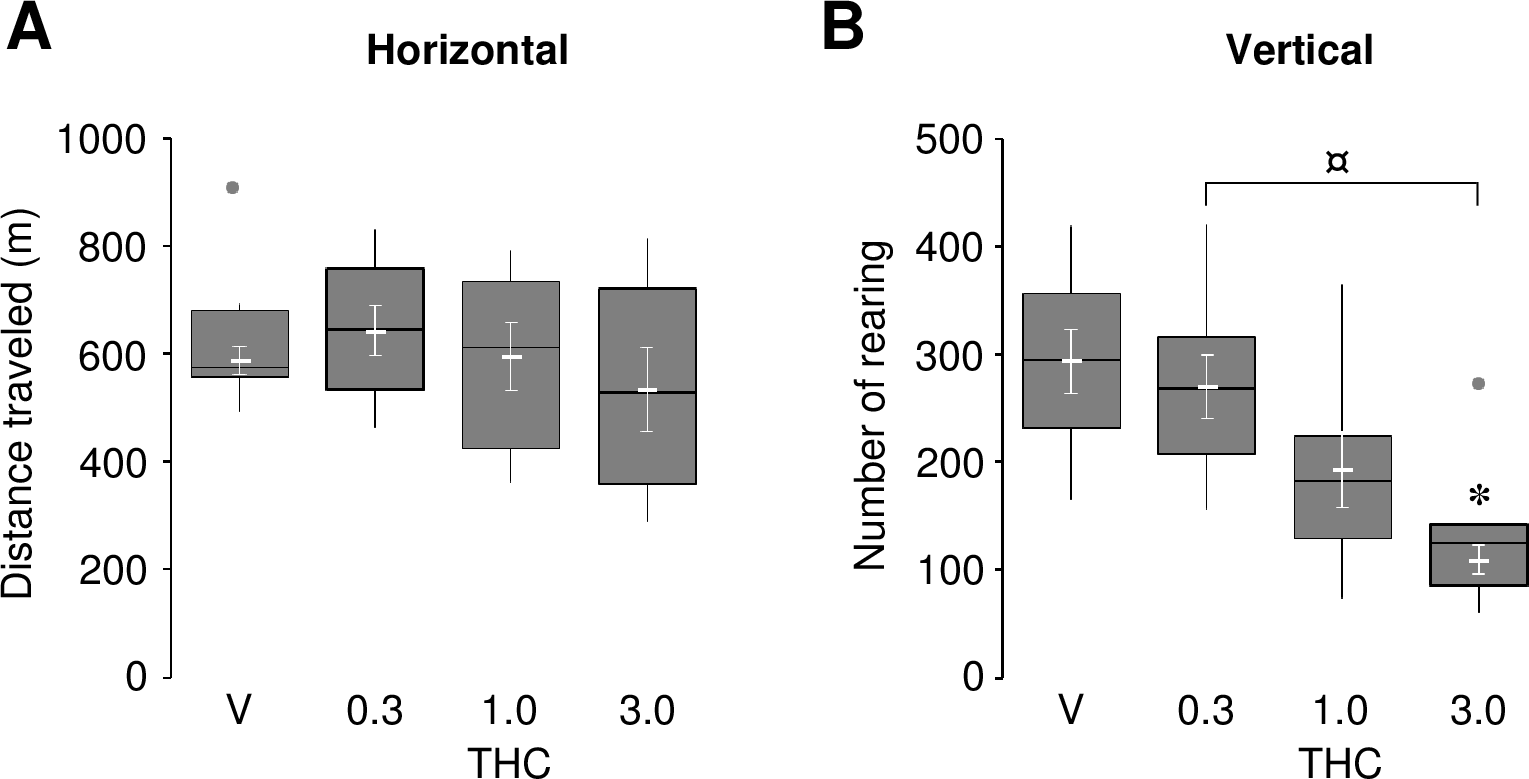

Supplement: S5 Fig — Distance traveled (A) and number of rearing (B) in the Actimot activity box in a novel environment following THC (0.3–3 mg/kg, i.p.) administration. The raw data for this figure are reported in the supplemental file (S1 Raw data) and are summarized here as boxplots computed using Carling’s modification (Carling, 2000); outliers are depicted as gray circles. Values (in white) are expressed as mean ± S.E.M. (n = 6–8 per group). ANOVA revealed no effect for horizontal (F3,25 = 0.65, P = 0.59), but one for vertical activity (F3,25 = 7.69, P < 0.001). * P < 0.001 compared to vehicle (V) control, ¤ P < 0.01 (Newman-Keuls post-hoc test). (TIF) [file pone.0230238.s005.tif]
