# Supplementary material for: Differential effects of Δ9-tetrahydrocannabinol dosing on correlates of schizophrenia in the sub-chronic PCP rat model
Source: PLoS One. 2020 Mar 12;15(3):e0230238. doi: 10.1371/journal.pone.0230238 (PMC7067407; doi:10.1371/journal.pone.0230238)

Gel 1

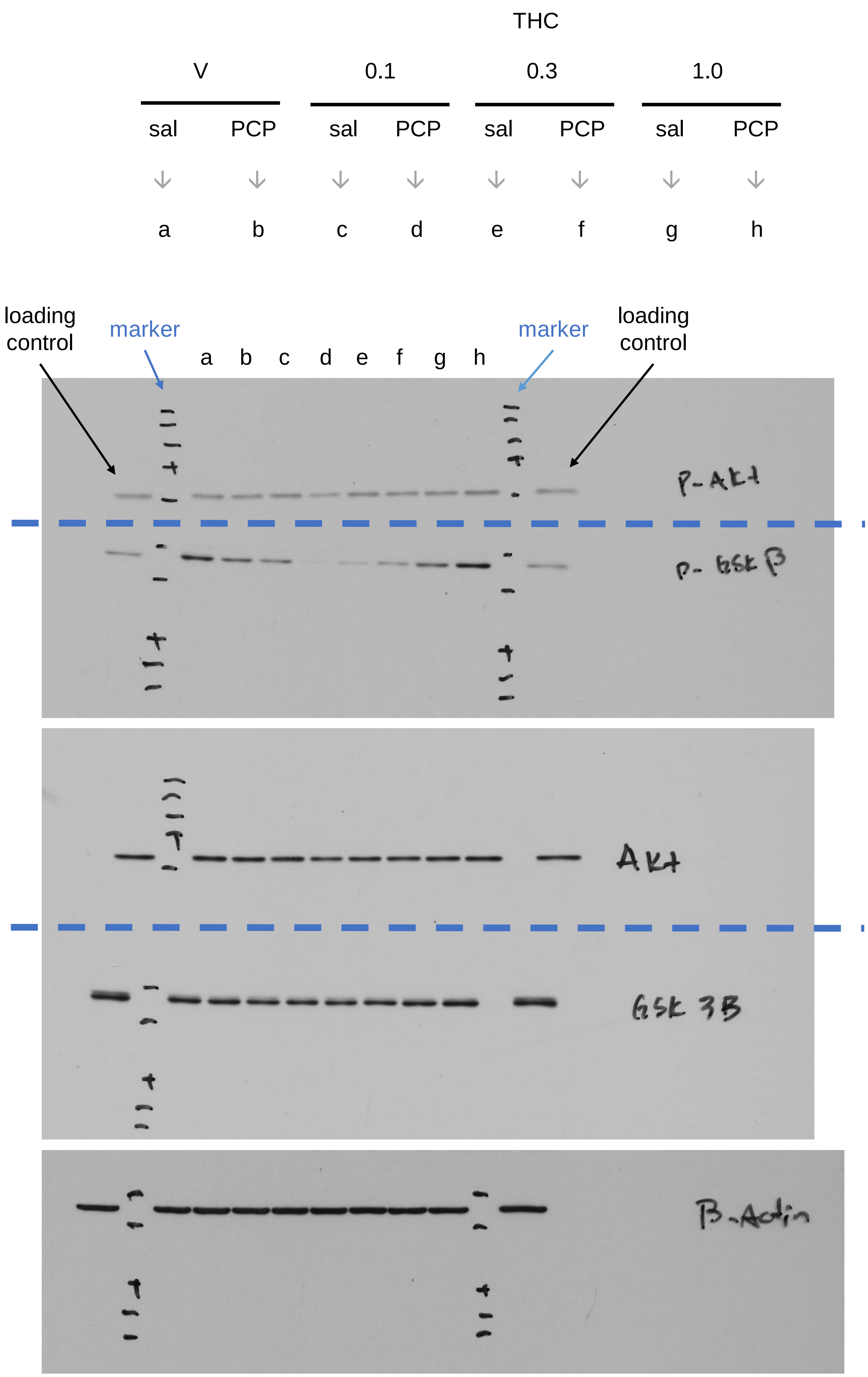
THC
V
0.1
0.3
1.0
sal
PCP
sal
PCP
sal
PCP
sal
PCP
a
b
c
d
e
f
g
h
loading control
marker
a b c d e f g h
marker
loading control
P-Akt
P- GSK β
Akt
GSK 3B
B-Actin

Gel 2

THC

V		0.1		0.3		1.0	
sal	PCP	sal	PCP	sal	PCP	sal	PCP
↓	↓	↓	↓	↓	↓	↓	↓
a	b	c	d	e	f	g	h

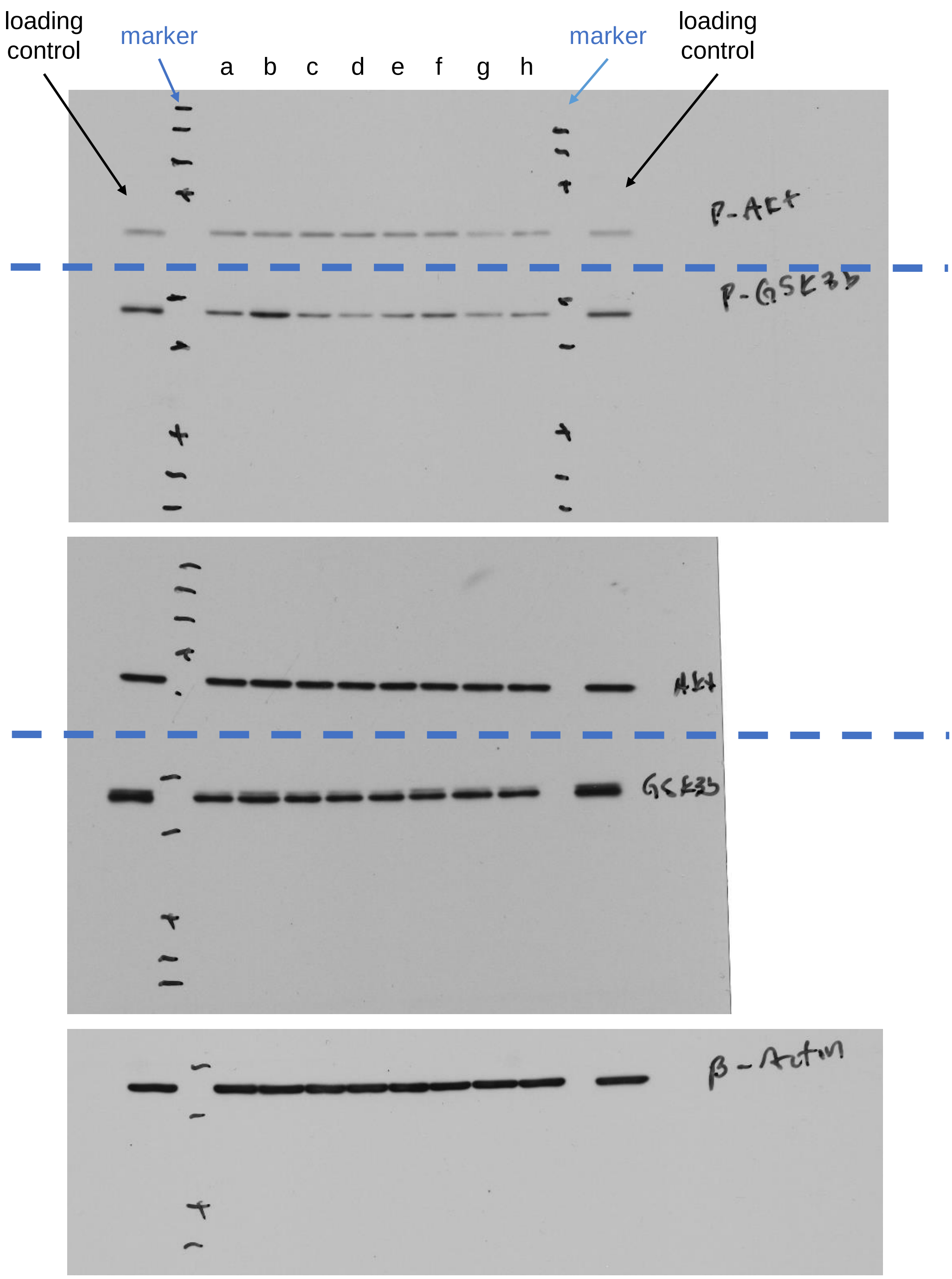

Gel 3

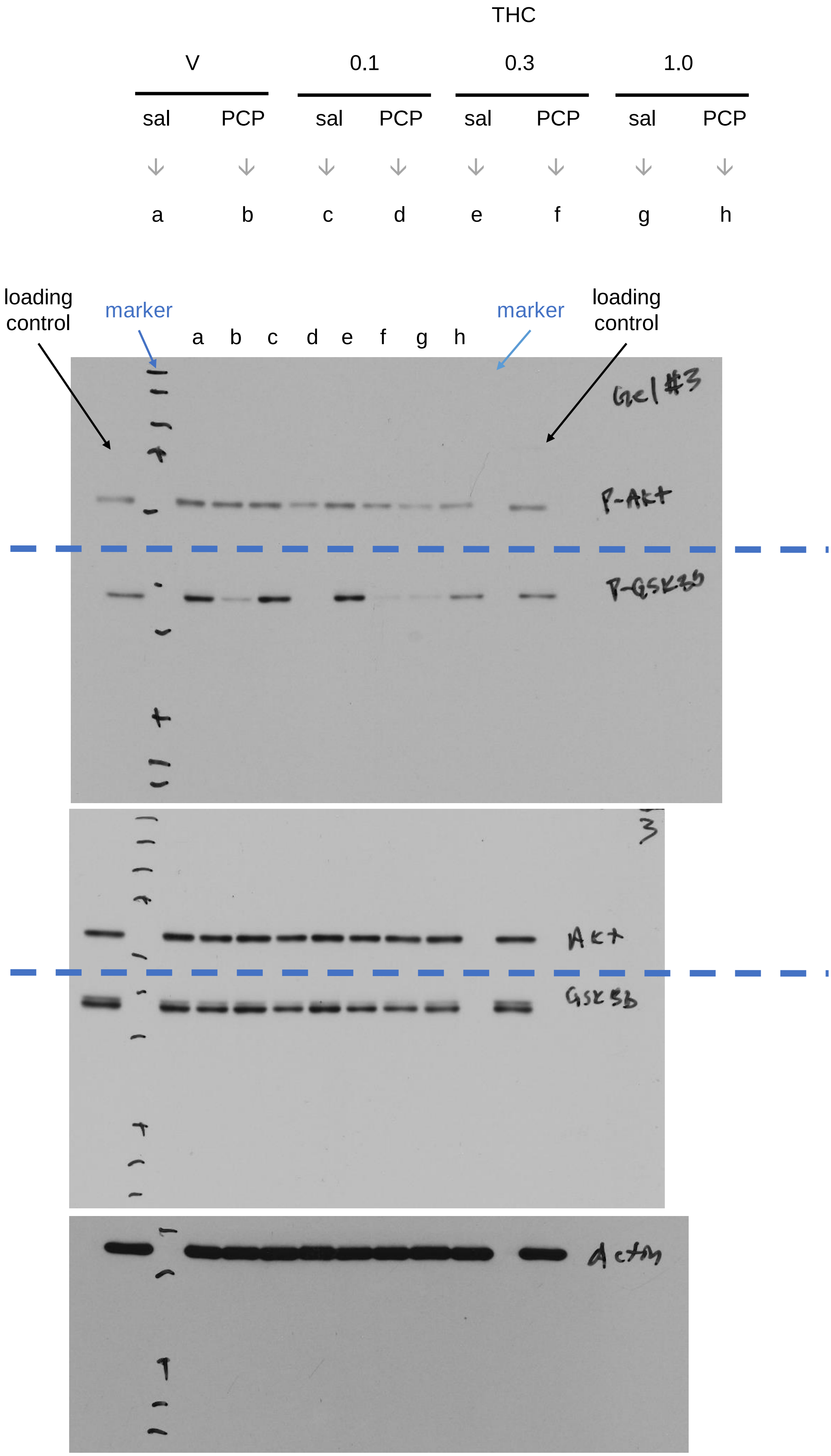
THC
V
0.1
0.3
1.0
sal
PCP
sal
PCP
sal
PCP
sal
PCP
a
b
c
d
e
f
g
h
loading control
marker
a b c d e f g h
marker
loading control
Gel #3
P-Akt
P-GSK3b
Akt
GSK3b
Actin

Gel 4

	THC						
V		0.1		0.3		1.0	
sal	PCP	sal	PCP	sal	PCP	sal	PCP
↓	↓	↓	↓	↓	↓	↓	↓
a	b	c	d	e	f	g	h

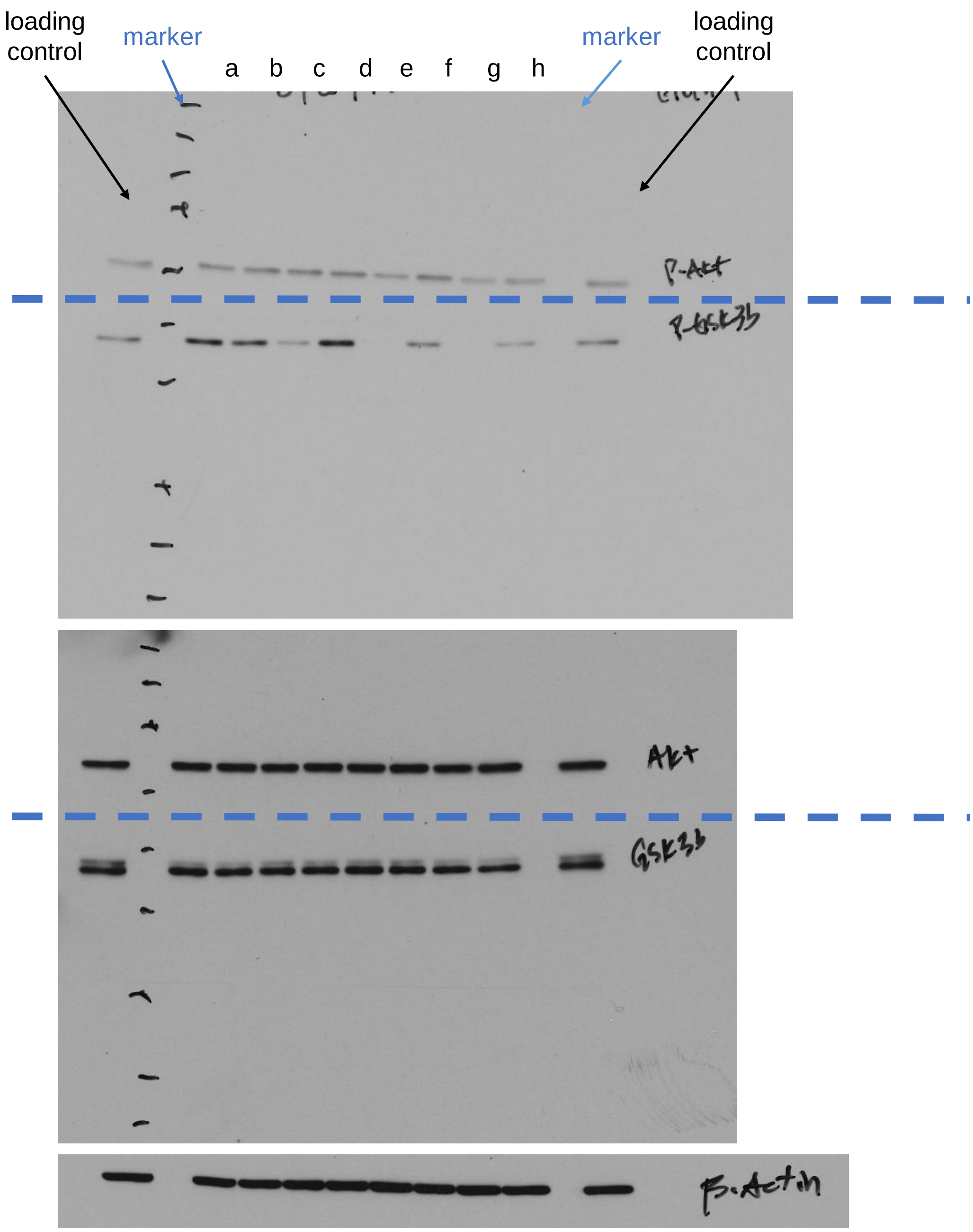

Gel 5

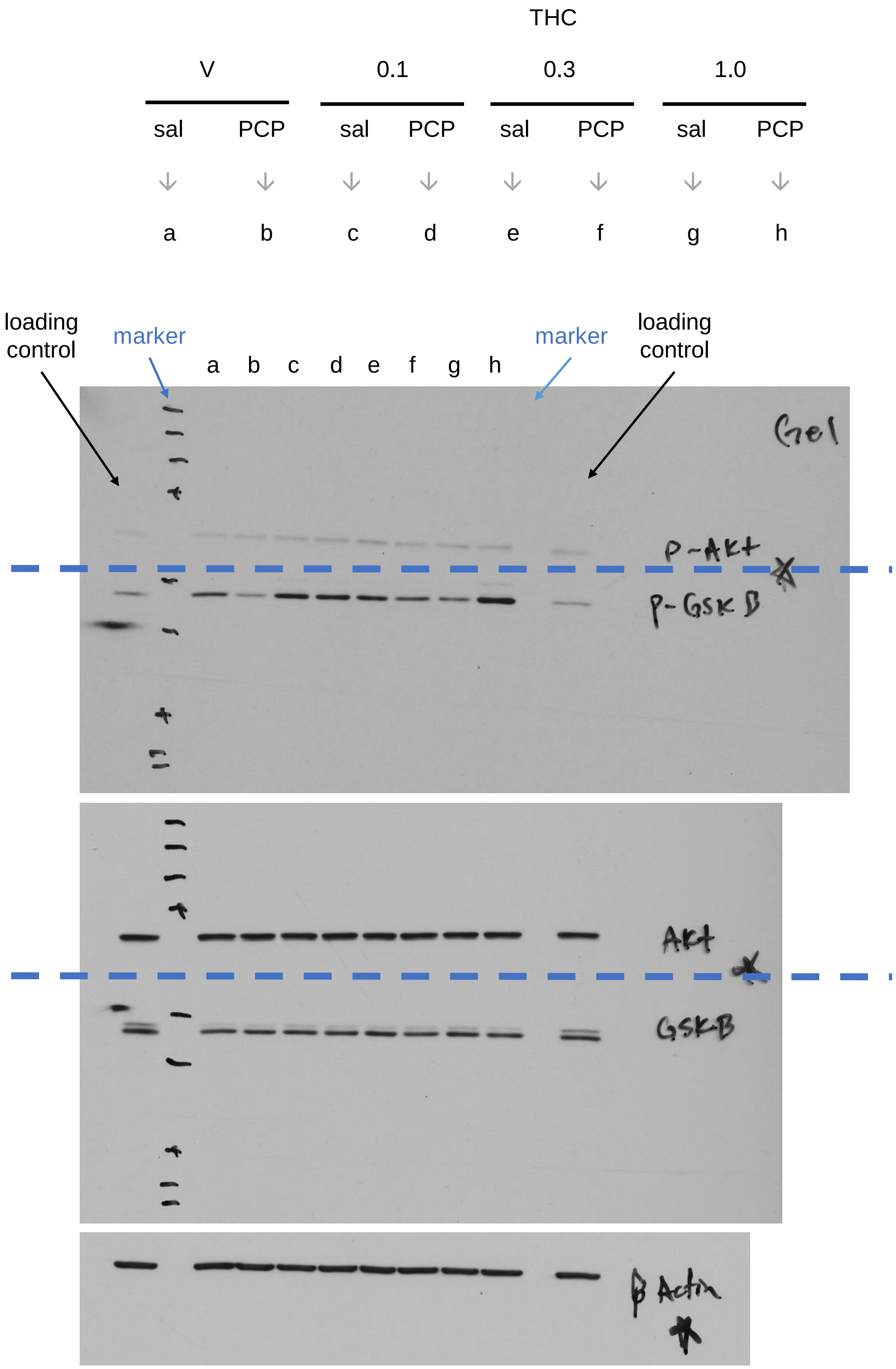
THC
V
0.1
0.3
1.0
sal
PCP
sal
PCP
sal
PCP
sal
PCP
a
b
c
d
e
f
g
h
loading control
marker
a b c d e f g h
marker
loading control
Gel
P-Akt
P-GSK B
Akt
GSKB
β Actin

Gel 6

		THC					
V		0.1		0.3		1.0	
sal	PCP	sal	PCP	sal	PCP	sal	PCP
↓	↓	↓	↓	↓	↓	↓	↓
a	b	c	d	e	f	g	h

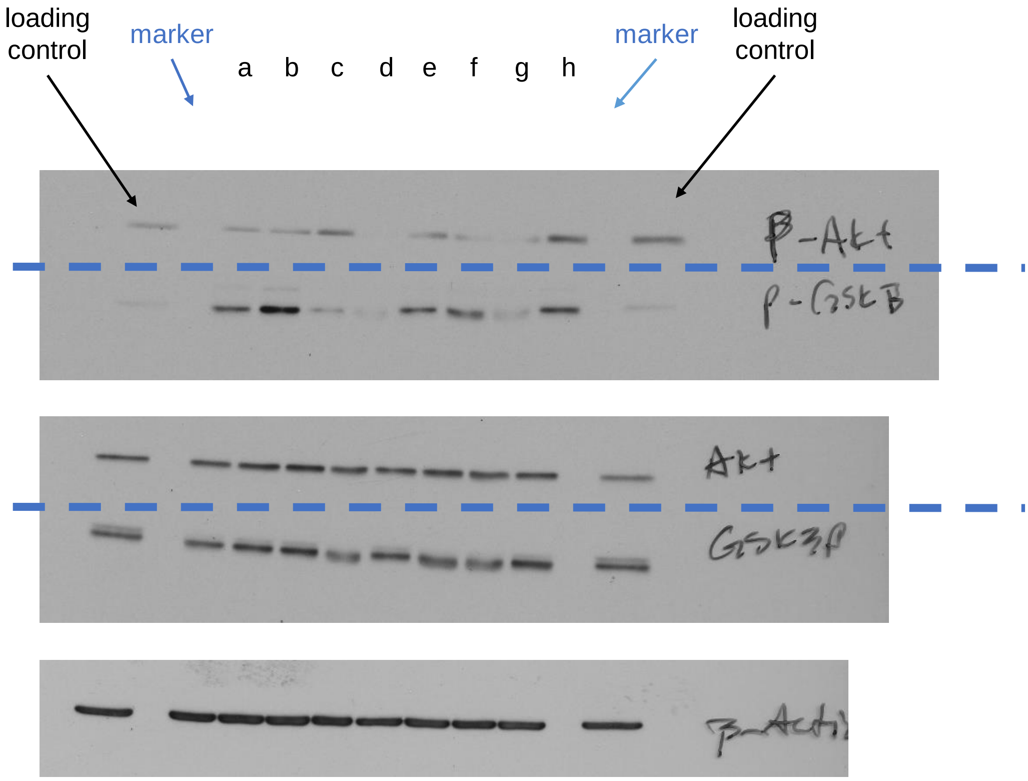

Gel 7

THC

V		0.1		0.3		1.0	
sal	PCP	sal	PCP	sal	PCP	sal	PCP
↓	↓	↓	↓	↓	↓	↓	↓
a	b	c	d	e	f	g	h

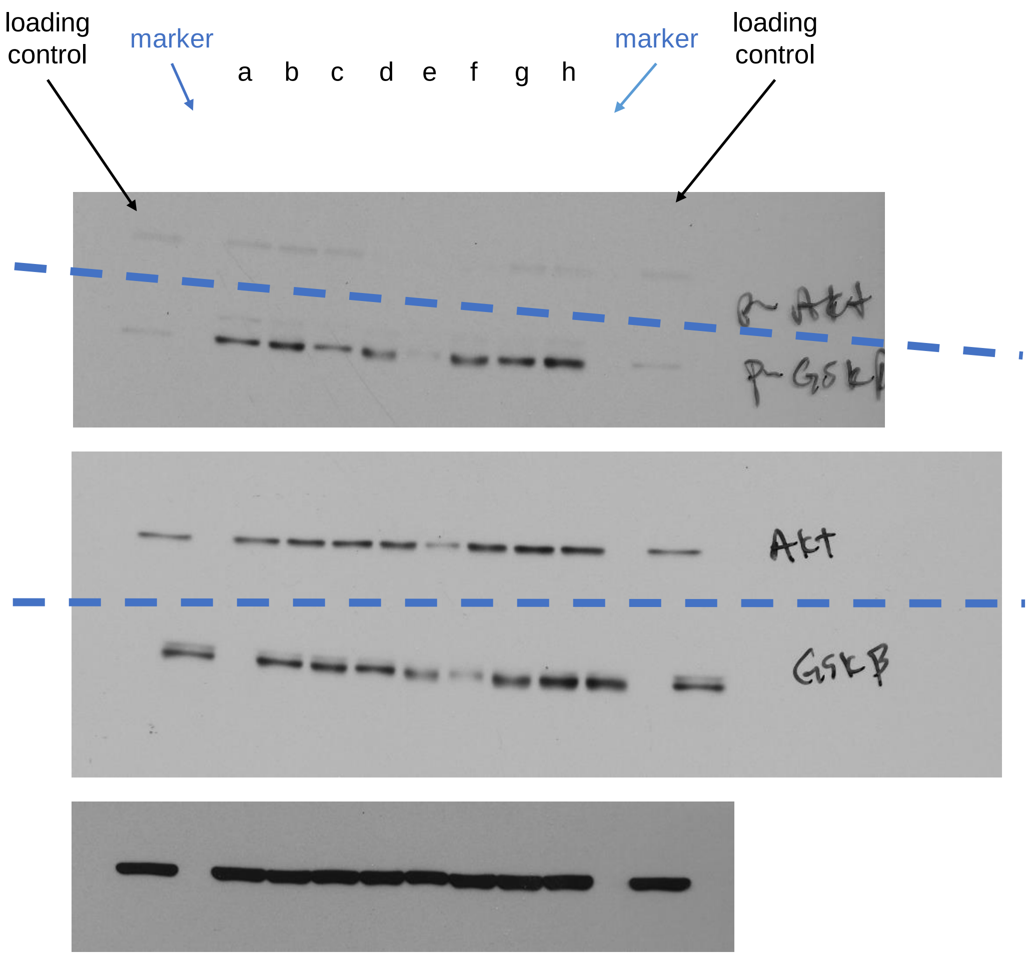

Supplement: S1 Raw images — (PDF) [file pone.0230238.s006.pdf]
